# Genes and Their Molecular Functions Determining Seed Structure, Components, and Quality of Rice

**DOI:** 10.1186/s12284-022-00562-8

**Published:** 2022-03-18

**Authors:** Pei Li, Yu-Hao Chen, Jun Lu, Chang-Quan Zhang, Qiao-Quan Liu, Qian-Feng Li

**Affiliations:** 1 Jiangsu Key Laboratory of Crop Genomics and Molecular Breeding/Jiangsu Key Laboratory of Crop Genetics and Physiology/State Key Laboratory of Hybrid Rice, College of Agriculture, Yangzhou University, Yangzhou, 225009 Jiangsu China; 2 Co-Innovation Center for Modern Production Technology of Grain Crops of Jiangsu Province/Key Laboratory of Plant Functional Genomics of the Ministry of Education, Yangzhou University, Yangzhou, 225009 Jiangsu China

**Keywords:** Rice grain quality, Seed structure, Seed size, Grain component, Starch, Protein, Gene cloning, Molecular function, Regulatory network

## Abstract

With the improvement of people's living standards and rice trade worldwide, the demand for high-quality rice is increasing. Therefore, breeding high quality rice is critical to meet the market demand. However, progress in improving rice grain quality lags far behind that of rice yield. This might be because of the complexity of rice grain quality research, and the lack of consensus definition and evaluation standards for high quality rice. In general, the main components of rice grain quality are milling quality (MQ), appearance quality (AQ), eating and cooking quality (ECQ), and nutritional quality (NQ). Importantly, all these quality traits are determined directly or indirectly by the structure and composition of the rice seeds. Structurally, rice seeds mainly comprise the spikelet hull, seed coat, aleurone layer, embryo, and endosperm. Among them, the size of spikelet hull is the key determinant of rice grain size, which usually affects rice AQ, MQ, and ECQ. The endosperm, mainly composed of starch and protein, is the major edible part of the rice seed. Therefore, the content, constitution, and physicochemical properties of starch and protein are crucial for multiple rice grain quality traits. Moreover, the other substances, such as lipids, minerals, vitamins, and phytochemicals, included in different parts of the rice seed, also contribute significantly to rice grain quality, especially the NQ. Rice seed growth and development are precisely controlled by many genes; therefore, cloning and dissecting these quality-related genes will enhance our knowledge of rice grain quality and will assist with the breeding of high quality rice. This review focuses on summarizing the recent progress on cloning key genes and their functions in regulating rice seed structure and composition, and their corresponding contributions to rice grain quality. This information will facilitate and advance future high quality rice breeding programs.

## Introduction

**Fig. 1:**
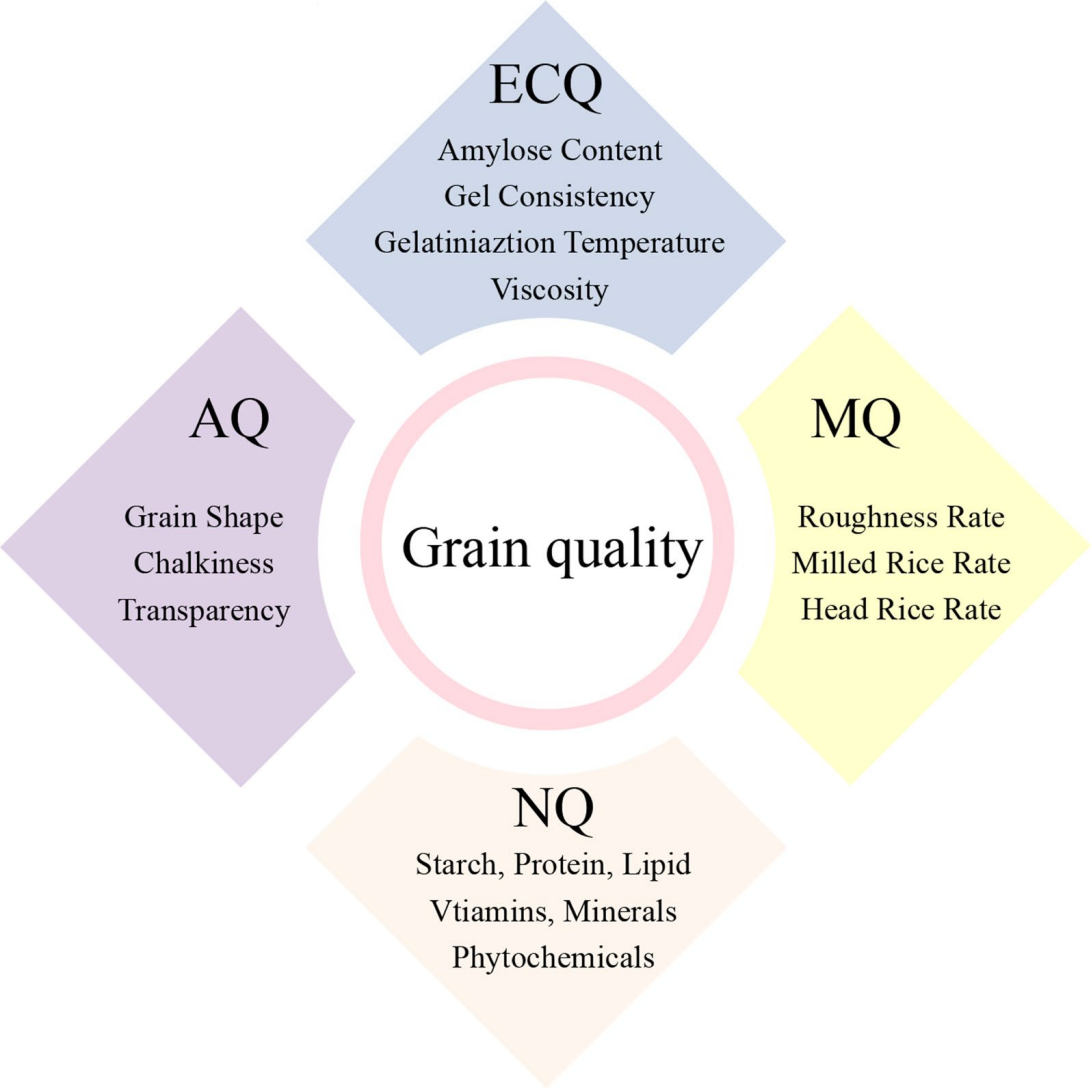
The scope of rice quality includes the following four parts, appearance quality (AQ), milling quality (MQ), nutritional quality (NQ), eating and cooking quality (ECQ)

Rice is the major food crop for more than half of the world's population. Breeding elite rice with high yield and quality are the major goals of crop geneticists and rice breeders. In recent decades, benefiting from the discovery and application of "green revolution gene" and "heterosis", rice yield has improved greatly (Evenson and Gollin 2003; Pingali 2012; Chen et al. 2019; Liu et al. 2020; Wu et al. 2020). However, the progress of rice quality-related research and breeding practices lag far behind that of rice yield. This might reflect the emphasis placed on rice yield in the past, the complexity of rice quality studies, and the lack of consensus definitions and evaluation standards of rice quality. As people's living standards and the worldwide rice trade improve, the demand for high-quality rice is increasing. Therefore, breeding high quality rice is critical to meet market demand. A series of physical and chemical indexes are used for the comprehensive evaluation of rice quality during the rice processing and cooking. In general, rice quality is divided into four main sections: milling quality (MQ), appearance quality (AQ), nutritional quality (NQ), and eating and cooking quality (ECQ) (Fig. 1) (Bao 2014). Rice MQ refers to the integrity of rice during processing, including the roughness rate, milled rice rate, and head rice rate. AQ usually includes grain shape, chalkiness, transparency, and other indicators. Rice NQ is influenced by the quantity and quality of starch, protein, vitamins, minerals, and other phytochemicals that are beneficial to human health. ECQ mainly reflects the characteristics and palatability of cooked rice. Amylose content (AC), gel consistency (GC), gelatinization temperature (GT), and the results gained using a rapid viscosity analyzer (RVA) are usually used as indirect indicators to estimate rice ECQ (Zhang et al. 2016).

**Fig. 2:**
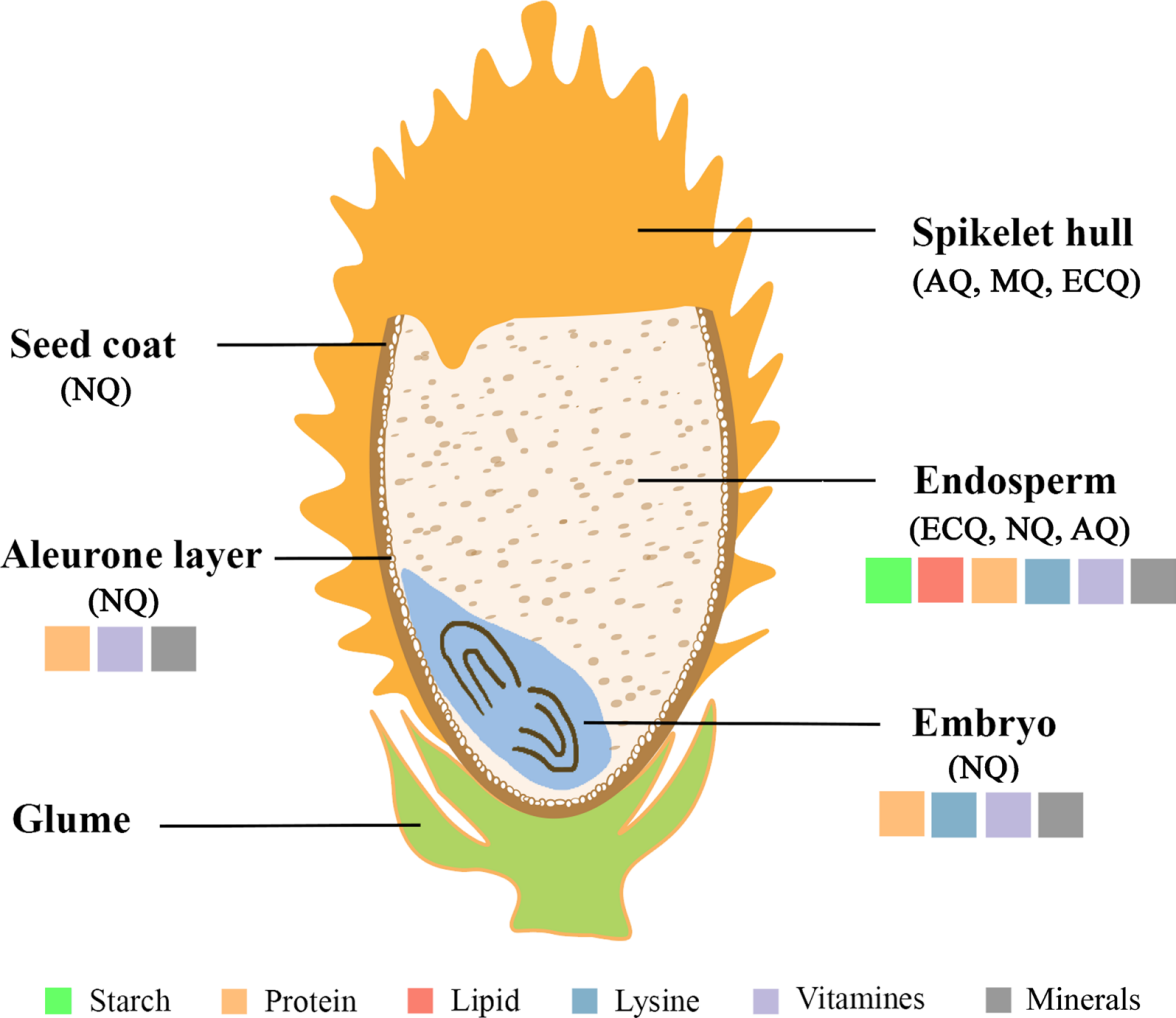
Overview of the main structure and components based on the longitudinal section of mature rice seeds. AQ, appearance quality; MQ, milling quality; NQ, nutritional quality; ECQ, eating and cooking quality. Not drawn in proportion

In general, rice grain quality should be closely correlated with rice seed characteristics. In brief, the rice seed structure mainly includes the spikelet hull, seed coat, aleurone layer, embryo, and endosperm (Fig. 2). The size, composition, and quality of these seed structures affect rice quality markedly. For example, in most cases, the size of the spikelet hull determines the grain size. Grain size is an important rice agronomic trait, which is not only a key element of rice yield, but also is a direct index for rice quality. Rice grain size includes grain length, width, thickness, and the ratio of length to width, which usually affects rice AQ, MQ, and ECQ. Recently, a series of grain size-related genes have been cloned successfully and functionally dissected (Li et al. 2019). The endosperm is the major edible part of rice seed. Therefore, the composition and proportion of endosperm components are the most important determinants of rice quality. In general, the rice endosperm includes starch, storage protein, lipids, minerals, and other trace elements. Among them, starch and protein are two major components of rice endosperm, accounting for approximately 80% and 10%, respectively, of the weight of milled rice (Wang et al. 2020a). As the largest constituent of rice endosperm, starch consists of two glucose polymers, amylose and amylopectin. The amylose content (AC) and amylopectin structure correlate tightly with rice ECQ (Leng et al. 2014). In addition, the arrangement of starch granules will affect rice AQ. For example, a looser arrangement of starch grains will form cavities inside or between the starch granules, thus decreasing the transparency of the rice grain and resulting in the so-called opaque or chalky endosperm (Zhang et al. 2017). As for proteins, the second largest component of rice endosperm, their content and amino acid composition will affect almost all aspects of rice quality (Duan and Sun 2005). Moreover, other substances in the endosperm, including lipids, free amino acids, minerals, vitamins, and other phytochemicals, albeit present in lower in quantities, are crucial for rice NQ. The other structures of rice seeds, including the embryo, seed coat, and aleurone layer, are rich in protein, fat, vitamins, and minerals. For example, functional metabolites, such as gamma aminobutyric acid (GABA), are enriched in embryo and aleurone layer compared with that in the endosperm (Pereira et al. 2021).

Each structure of the rice seed correlates with various rice quality traits, and rice grain development is directly controlled by many genes; hence, cloning and dissecting these quality-genes will aid the breeding of high quality rice. Therefore, this review focused on summarizing the recently cloned key genes involved in regulating rice seed structure and composition, and their roles in modulation of rice qualities (Table 1), which will facilitate and advance future high quality rice breeding programs.

**Table 1:** List of genes involved in controlling rice seed structures and grain qualities

Gene ID	Gene name	Protein category	Effect the quality	References
*Spikelet hull & grain size & grain quality*				
G protein pathway				
Os03g0407400	*GS3*	Gγ subunit	Grain length (AQ)	Fan et al. (2006) Mao et al. (2010) Sun et al. (2018a)
Os09g0441900	*DEP1/qPE9*	Gγ subunit	Grain length (AQ)	Zhou et al. (2009) Huang et al. (2009)
Os03g0635100	*RGG1*	Gγ subunit	Grain length (AQ)	Tao et al. (2020)
Os02g0137800	*RGG2*	Gγ subunit	Grain length (AQ)	Miao et al. (2019)
Os08g0456600	*GGC2*	Gγ subunit	Grain length (AQ)	Sun et al. (2018a)
Os05g0333200	*D1/RGA1*	Gα subunit	Grain length (AQ)	Fujisawa et al. (1999) Ashikari et al. (1999) Sun et al. (2018a)
Os03g0669200	*RGB1*	Gβ subunit	Grain length (AQ)	Utsunomiya et al. (2011) Zhang et al. (2021b)
The ubiquitin–proteasome pathway				
Os02g0244100	*GW2*	E3 ubiquitin ligase	Grain width (AQ)	Song et al. (2007)
Os02g0244300	*LG1/OsUBP15*	Ubiquitin specific protease	Grain width (AQ)	Shi et al. (2019)
Os03g0232600	*TUD1*	U-box E3 ubiquitin ligase	Grain length (AQ)	Hu et al. (2013)
Os02g0512400	*OsGRX8/WG1*	CC-type glutaredoxin	Grain length, grain width (AQ)	Hao et al. (2021)
Os06g0265400	*OsbZIP47*	bZIP transcription factor	Grain width (AQ)	Hao et al. (2021)
Os08g0537800	*WTG1/OsOTUB1*	Deubiquitinating enzyme	Grain width, grain thickness (AQ)	Huang et al. (2017)
Os06g0650300	*GW6a/OslHAT1*	GNAT-like Protein	Grain length (AQ)	Song et al. (2015) Gao et al. (2021)
Os05g0551000	*CLG1/OsHRZ2*	RING E3 ubiquitin ligase	Grain length (AQ)	Yang et al. (2021b)
Mitogen-activated protein kinase (MAPK) signaling				
Os02g0787300	*OsMKK4/SMG1*	Mitogen-activated protein kinase kinase	Grain length, grain width (AQ)	Duan et al. (2014) Guo et al. (2018)
Os06g0154500	*OsMAPK6/DSG1*	Mitogen activated protein kinase	Grain length, grain width (AQ)	Liu et al. (2015b)
Os04g0559800	*SMG2/OsMKKK10*	Mitogen activated protein kinase kinase kinase	Grain length, grain width (AQ)	Xu et al. (2018)
Os01g0699500	*OsMKKK70*	Mitogen activated protein kinase kinase kinase	Grain length, grain width (AQ)	Liu et al. (2021)
Os01g0699600	*OsMKKK62*	Mitogen activated protein kinase kinase kinase	Grain length, grain width (AQ)	Liu et al. (2021)
Os05g0115800	*OsMKP1/GSN1*	MAPK phosphatase	Grain length, grain width (AQ)	Guo et al. (2018) Xu et al. (2018)
Phytohormone perception and homeostasis				
Brassinosteroids				
Os03g0602300	*BRD1/OsDWARF*	Brassinosteroid biosynthetic enzyme	Grain length (AQ)	Hong et al. (2002) Mori et al. (2002)
Os10g0397400	*BRD2*	Brassinosteroid biosynthetic enzyme	Grain length (AQ)	Hong et al. (2005)
Os04g0469800	*D11/* CYP724B1	Brassinosteroid biosynthetic enzyme	Grain length (AQ)	Tanabe et al. (2005) Zhu et al. (2015) Wu et al. (2016b) Zhou et al. (2017c)
Os05g0187500	*GW5/GSE5/qGW5*	Calmodulin binding protein	Grain width, chalkiness (AQ) Milled rice rate (MQ)	Weng et al. (2008) Shomura et al. (2008) Liu et al. (2017) Duan et al. (2017)
Os05g0207500	*OsGSK2*	GSK3/SHAGGY-like kinase	Grain length (AQ)	Tong et al. (2012)
Os02g0236200	*OsGSK3*	GSK3/SHAGGY-like kinase	Grain length (AQ)	Gao et al. (2019)
Os07g0580500	*OsBZR1*	Transcription factor in BR pathway	Grain length, grain width, grain thickness (AQ)	Zhu et al. (2015)
Os02g0517531	*LARGE1/ OML4*	MEI2-like protein	Grain length, grain width (AQ)	Lyu et al. (2020)
Os06g0127800	*DLT/D62/GS6*	GRAS family protein	Grain width (AQ)	Tong et al. (2009) Sun et al. (2013)
Os05g0158500	*GS5*	Putative serine carboxypeptidase	Grain width (AQ)	Li et al. (2011) Xu et al. (2015)
Os05g0343400	*OsWRKY53*	WRKY transcription factor	Grain length, grain width (AQ)	Tian et al. (2017)
Os08g0174700	*OsBAK1*	BRI1-associated receptor kinase	Grain length, grain width (AQ)	Li et al. (2009)
Os03g0646900	*GL3.1/GL3-1/qGL3/OsPPKL1*	Ser/Thr phosphatase	Grain width (AQ)	Hu et al. (2012) Qi et al. (2012) Zhang et al. (2012a) Gao et al. (2019)
Os04g0674500	*OsmiR396d*	MicroRNA	Grain length, grain width (AQ)	Miao et al. (2020)
Os02g0701300	*GS2/GL2/OsGRF4/PT2/ LGS1*	Growth-regulating factor	Grain length, grain width (AQ)	Che et al. (2015) Duan et al. (2015) Hu et al. (2015) Sun et al. (2016) Li et al. (2016) Sun et al. (2016)
Os05g0458600	*OsLAC*	Laccase-like protein	Grain length, grain width (AQ)	Zhang et al. (2013) Zhong et al. (2020)
Os02g0169400	*OsAGO17*	Argonaute protein	Grain length, grain width (AQ)	Zhong et al. (2020)
Os09g0448500	*GS9*	Transcriptional activator	Grain width, chalkiness (AQ)	Zhao et al. (2018)
Os01g0718300	*OsBRI1/D61*	BR receptor kinase	Grain length, grain width (AQ)	Yamamuro et al. (2000)
Os01g0226700	*OFP1*	OVATE family protein	Grain length, grain width (AQ)	Xiao et al. (2017)
Os01g0732300	*OFP3*	OVATE family protein	Grain length, grain width (AQ)	Xiao et al. (2020)
Os01g0864000	*OFP8*	OVATE family protein	Grain length, grain width (AQ)	Yang et al. (2016a, b)
Os05g0324600	*OFP19*	OVATE family protein	Grain length (AQ)	Yang et al. (2018a)
Os05g0477200	*OFP22*	OVATE family protein	Grain length, grain width (AQ)	Chen et al. (2021)
*Os10g0515400*	*GW10*	Cytochrome P450 subfamily protein	Grain length, grain width (AQ)	Zhan et al. (2021)
Os07g0175100	*POW1*	Homeodomain-like protein	Grain length, grain width (AQ)	Zhang et al. (2021c)
Auxin				
Os06g0623700	*TGW6*	Protein with IAA-glucose hydrolase activity	Grain length, grain width (AQ)	Ishimaru et al. (2013) Akabane et al. (2021) Kabir and Nonhebel (2021)
Os03g0175800	*BG1*	Positive regulator of auxin response and transport	Grain length, grain width (AQ)	Liu et al. (2015a)
Os03g0841800	q*TGW3/GL3.3/qGL6*	GSK3/SHAGGY-like kinase	Grain length, grain width, grain thickness (AQ)	Hu et al. (2018) Xia et al. (2018) Ying et al. (2018)
Os02g0164900	*OsARF6*	Auxin response factor	Grain length (AQ)	Qiao et al. (2021)
Os05g0447200	*OsAUX3/qGL5*	Auxin influx carrier	Grain length (AQ)	Qiao et al. (2021)
GA				
Os06g0266800	*OsGSR1/GW6/OsGASR7*	GA-stimulated protein	Grain length, grain width (AQ)	Shi et al. (2020)
Cytokinin				
Os01g0680200	*OsPUP4/ BG3*	Purine permease	Grain length, grain width, grain thickness (AQ)	Xiao et al. (2019) Yin et al. (2020)
Os04g0615700	*OsAGO2*	AGO family protein	Grain length (AQ)	Yin et al. (2020)
Transcriptional regulatory factors				
SQUAMOSA promoter binding protein-like (SPL) family				
Os07g0505200	*OsSPL13/GLW7*	Squamosa promoter-binding-like protein	Grain length, grain width (AQ)	Si et al. (2016) Segami et al. (2017)
Os11g0247300	*SRS5/TID1*	Alpha-tubulin protein	Grain length, grain width (AQ)	Sunohara et al. (2009) Segami et al. (2012) Segami et al. (2017)
Os08g0531600	*qGW8/OsSPL16*	Squamosa promoter binding-like protein	AC, GC (ECQ)) Protein content (NQ)	Wang et al. (2012) Wang et al. (2015a)
Os07g0603300	*GL7/GW7*	TRM-containing protein	Grain length, grain width, chalkiness (AQ) AC, GC (ECQ)) Protein content (NQ)	Wang et al. (2015c)
APETALA2-type (AP2) transcription factors				
Os05g0389000	*SMOS1/SHB*	AP2-type transcription factor	Grain length, grain width (AQ)	Aya et al. (2014)
Os07g0235800	*SSH1*	AP2-like transcription factor	Grain length, grain width (AQ)	Jiang et al. (2019)
Basic helix-loop-helix (bHLH) family				
Os04g0350700	*An-1*	Basic helix-loop-helix protein	Grain length (AQ)	Luo et al. (2013)
Os03g0171300	*PGL1*	Atypical non-DNA-binding bHLH protein	Grain length (AQ)	Heang and Sassa (2012)
Other transcription factors				
Os06g0666100	*GL6/SG6*	PLATZ transcription factor	Grain length (AQ)	Wang et al. (2019) Zhou and Xue (2020)
Os03g0215400	*OsMADS1/qLGY3*	MADS-domain transcription factor	Grain length (AQ)	Liu et al. (2018b, a) Yu et al. (2018)
Os03g0333200	*FLR1*	Receptor-like kinase	Grain width, chalkiness (AQ)	Wang et al. (2021)
Os01g0769700	*FLR2*	Receptor-like kinase	Grain length (AQ)	Wang et al. (2021)
Other functional proteins				
Os08g0485500	*GAD1*	Secretory signal peptide	Grain length (AQ)	Jin et al. (2016)
ORGLA04G0254300	*GL4*	Myb like protein similar to SH4/SHA1	Grain length (AQ)	Wu et al. (2017)
Os07g0214300	*RAG2*	16-kDa α-amylase/trypsin inhibitor	Grain length, grain width (AQ) Protein content, lipid content (NQ)	Zhou et al. (2017b)
*Endosperm components and grain quality*				
Starch				
Os06g0133000	*Wx*	Granule-bound starch synthase	AC, GC, GT (ECQ) Transparency, chalkiness (AQ) Digestion, RS (NQ)	Wang et al. (2010) Zhang et al. (2019a) Huang et al. (2020) Zhang et al. (2021a) Zhou et al. (2021a)
Os02g0744700	*OsSSIIb/SSII-2*	Soluble starch synthase	AC, GC, GT (ECQ) Transparency (AQ) Protein content (NQ)	Li et al. (2018a, b) Xu et al. (2020)
Os06g0229800	*OsSSIIa/SSII-3/ALK*	Soluble starch synthase	GT (ECQ)	Umemoto et al. (2002) Gao et al. (2003) Chen et al. (2020) Zhang et al. (2020)
Os08g0191433	*OsSSIIIa/FLO5*	Soluble starch synthase	Chalkiness (AQ) AC, GC (ECQ) Digestion, RS (NQ)	Fujita et al. (2007) Zhou et al. (2016)
Os02g0528200	*OsSBEIIb/SBE3*	Starch branching enzyme	Chalkiness (AQ) AC, GC (ECQ) Digestion, RS (NQ)	Zhu et al. (2012) Baysal et al. (2020) Miura et al. (2021)
Os07g0182000	*OsbZIP58/ RISBZ1*	bZIP transcription factor	Chalkiness (AQ) AC (ECQ)	Yamamoto et al. (2006) Kawakatsu et al. (2009) Wang et al. (2013)
Os02g0725900	*OsNF-YB1*	Component of the NF-Y/HAP transcription factor complex	Grain length, grain width, chalkiness (AQ) AC (ECQ) Protein content, lipid content (NQ)	Bello et al. (2019) Xu et al. (2021)
Os10g0191900	*OsNF-YC12/OsNF-YC11*	Nuclear transcription factor Y subunit C	Grain length, grain width, chalkiness (AQ) AC (ECQ) Protein content, lipid content (NQ)	Bello et al. (2019) Xiong et al. (2019)
Os12g0189500	*OsYUC11*	Flavin-containing monooxygenase	Grain length, grain width, chalkiness (AQ) Protein content, lipid content (NQ)	Xu et al. (2021)
Os02g0682200	*OsMADS6/AFG1*	MADS-box protein	Grain length, grain width (AQ) AC, GC (ECQ) Protein content (NQ)	Zhang et al. (2010)
Os02g0170300	*OsMADS29*	MADS-box protein	Shrunken seeds (AQ) AC (ECQ)	Yin and Xue (2012) Nayar et al. (2013)
Os08g0531700	*OsMADS7/OsMADS45*	MADS-box protein	AC (ECQ)	Zhang et al. (2018a)
Os01g0104500	*OsNAC20/ONAC020*	NAC transcription factor	Grain width, grain thickness (AQ) Starch content (ECQ) Protein content (NQ)	Wang et al. (2020a)
Os01g0393100	*OsNAC26/ONAC026*	NAC transcription factor	Grain width, grain thickness (AQ) Starch content (ECQ) Protein content (NQ)	Wang et al. (2020a)
Os03g0686900	*FLO6*	CBM48 domain-containing protein	Transparency, chalkiness (AQ) Starch content (ECQ) Protein content, lipid content (NQ)	Peng et al. (2014)
Os07g0688100	*FLO18*	PPR protein	Transparency, chalkiness (AQ) AC, starch content (ECQ) Protein content, lipid content(NQ)	Yu et al. (2021)
Os03g0168400	*FLO10*	PPR protein	Transparency (AQ) Protein content, lipid content (NQ)	Wu et al. (2019)
Os08g0290000	*FGR1*	Nuclear-localized PPR protein	Grain thickness, transparency (AQ) AC, starch content (ECQ)	Hao et al. (2019)
Os03g0728200	*FLO14*	Nuclear-localized PPR protein	Chalkiness (AQ)	Xue et al. (2019)
Os07g0181000	*OsPK2/PKpα1*	Plastidic pyruvate kinase	Starch content (ECQ) Protein content, lipid content (NQ)	Cai et al. (2018)
Protein				
Os10g0400200	*OsGluA2/qGPC-10*	Glutelin type-A2 precursor	GC, starch content (ECQ) Protein content (NQ)	Yang et al. (2019)
Os05g0499100	*Glb1*	26 kDa α-gloubulin	Seed storage protein (NQ)	Wu et al. (1998)
Os01g0762500	*GluA1*	Glutelin type-A	Seed storage protein (NQ)	Qu et al. (2008)
Os02g0249800	*GluB1a*	Glutelin type-B	Seed storage protein (NQ)	Wu et al. (1998)
Os02g0249900	*GluB1b*	Glutelin type-B	Seed storage protein (NQ)	Wu et al. (1998)
Os02g0268300	*GluB4*	Glutelin type-B	Seed storage protein (NQ)	Qu and Takaiwa (2004)
Os02g0268100	*GluB5*	Glutelin type-B	Seed storage protein (NQ)	Qu et al. (2008)
Os02g0722400	*OsAAP10*	Amino acid permease	AC, RVA (ECQ) Protein content (NQ)	Wang et al. (2020b)
Os12g0631100	*OsRab5a/gpa1*	Small GTPase	Chalkiness (AQ) Protein content (NQ)	Ren et al. (2020)
Os06g0643000	*OsGPA5*	Rab5a effector	Chalkiness (AQ) Protein content (NQ)	Ren et al. (2020)
Os03g0835800	*GPA3*	Regulator of post-Golgi vesicular Traffic	AC (ECQ) Protein content (NQ)	Ren et al. (2014)
Os08g0127100	*OsHT/OsLHT1*	Amino acid transporter	AC, GC (ECQ) Protein content (NQ)	Guo et al. (2020a) Guo et al. (2020b)
Os02g0252400	*RPBF/OsDof3*	Prolamin box binding factor	Starch content (ECQ) Protein content, lipid content (NQ)	Kawakatsu et al. (2009)
Os07g0668600	*OsGZF1*	CCCH‐type zinc‐finger transcription factor	Glutelin content (NQ)	Chen et al. (2014)
Lipid/fat				
Os02g0716500	*OsFAD2-1*	Fatty acid desaturase	Lipid content (NQ)	Shi et al. (2012)
Os12g0104400	*OsFAD3*	Fatty acid desaturase	Lipid content (NQ)	Liu et al. (2012)
Os03g0369100	*OsLTP36*	Lipid transfer protein	Lipid content (NQ)	Wang et al. (2015b)
Os04g0436100	*OsACOT*	Acyl-CoA thioesterase	Grain length, grain width (AQ) Lipid content (NQ)	Zhao et al. (2019)
Os08g0110700	*FSE1*	Phospholipase-like protein	Lipid content (NQ)	Long et al. (2018)
Os01g0172400	*OsPLDα1*	Phospholipase Dα	Phytic acid content (NQ) AC,GT, setback viscosity, viscosity profiles (ECQ)	Khan et al. (2019) Khan et al. (2020)
Lysine (Amino acids)				
Os01g0927900	*AK2*	Aspartate kinase	Lysine content (NQ)	Yang et al. (2020)
Os04g0574800	*DHPS*	Dihydrodipicolinate synthase	Lysine content (NQ)	Yang et al. (2020) Yang et al. (2021a)
Os02g0783700	*OsLKR/SDH*	Lysine ketoglutarate reductase	Lysine content (NQ)	Yang et al. (2020)
Fe, Zn (Minerals)				
Os08g0207500	*OsZIP4*	Zinc-regulated transporter	Zinc content (NQ)	Ishimaru et al. (2007b)
Os05g0472700	*OsZIP5*	Zinc-regulated transporter	Zinc content (NQ)	Lee et al. (2010a)
Os07g0232800	*OsZIP8*	Zinc-regulated transporter	Zinc content (NQ)	Lee et al. (2010b)
Os03g0667500	*OsIRT1*	Iron-regulated transporter	Iron content (NQ)	Lee and An (2009)
Os03g0667300	*OsIRT2*	Iron-regulated transporter	Iron content (NQ)	Nakanishi et al. (2006)
Os11g0106700	*OsFER1*	Rice ferritin protein	Iron content (NQ)	Stein et al. (2009)
Os12g0106000	*OsFER2*	Rice ferritin protein	Iron content (NQ)	Stein et al. (2009)
Os03g0307300	*OsNAS1*	Nicotianamine synthase	Iron content (NQ)	Inoue et al. (2003)
Os03g0307200	*OsNAS2*	Nicotianamine synthase	Iron content (NQ)	Inoue et al. (2003)
Os07g0689600	*OsNAS3*	Nicotianamine synthase	Iron content (NQ)	Inoue et al. (2003)
Os04g0542200	*OsYSL9*	Probable metal-nicotianamine transporter	Iron content (NQ)	Senoura et al. (2017)
Os04g0463400	*OsVIT1*	Vacuolar membrane transporter	Iron content (NQ)	Zhang et al. (2012b)
Os09g0396900	*OsVIT2*	Vacuolar membrane transporter	Iron content (NQ)	Zhang et al. (2012b)
Phytochemicals				
Os08g0424500	*OsBadh2*	Betaine aldehyde dehydrogenasea	2-AP content (ECQ)	Chen et al. (2006) Chen et al. (2008) Kovach et al. (2009) Bradbury et al. (2010) Hui et al. (2021)
Embryo and grain quality				
Os07g0603700	*OsGE*	Cytochrome P450	Embryo size (NQ)	Nagasawa et al. (2013)
Os04g0447800	*OsGAD2*	Glutamate decarboxylase	GABA content (NQ)	Akama and Takaiwa (2007) Akama et al. (2009)
Os02g0112900	*OsGABA-T*	γ-aminobutyrate transaminase	GABA content (NQ)	Shimajiri et al. (2013)
Os03g0236200	*OsGAD3*	Glutamate decarboxylase	GABA content (NQ)	Akama et al. (2020)
Aleurone layer and grain quality				
Os05g0509700	*TA1/OsmtSSB1*	Mitochondrion-targeted single-stranded DNA binding protein	Thick aleurone (NQ)	Li et al. (2021)
Os01g0218032	*TA2-1/OsROS1*	DNA demethylase	Thick aleurone (NQ)	Liu et al. (2018a)
Seed coat and grain quality				
Os07g0211500	*Rc*	bHLH protein	Anthocyanin content (NQ)	Sweeney et al. (2006)
Os01g0633500	*Rd/OsDFR*	Dihydroflavonol reductase	Anthocyanin content (NQ)	Furukawa et al. (2007)
Os04g0557500	*Kala4/OsB2*	bHLH transcription factor	Anthocyanin content (NQ)	Oikawa et al. (2015)
Os06g0205100	*OsC1*	MYB transcriptional activator	Anthocyanin content (NQ)	Sun et al. (2018b)
Os02g0682500	*OsTTG1*	WD40 repeat protein	Anthocyanin content (NQ)	Yang et al. (2021c)

## Genes Regulating Spikelet Hull Development and Their Roles in Grain Qualities

In general, rice MQ correlates negatively with the grain length, grain length–width ratio, and grain length–thickness ratio, and positively with the grain width, grain thickness, and grain width–thickness ratio. Hence, reducing the length and increasing the width and thickness of rice grains are beneficial to improving rice MQ. Rice AQ is usually related to grain size, chalkiness, and transparency. The chalky grain percentage correlates positively with the 1000-grain weight, grain width, grain thickness, and grain width–thickness ratio. There is a significant positive correlation between the grain width and chalkiness. The grain filling rate of a wide grain is too fast, which leads to a looser arrangement of granules and the subsequent formation of chalkiness. Chalkiness is a negative element of rice quality, the higher the chalkiness, the poorer AQ, MQ, and ECQ of rice (Cheng et al. 2005; Yamakawa et al. 2007). For example, chalky rice breaks more easily during processing, resulting in less head rice and a decreased MQ. In contrast, smaller or slender seeds have less chalkiness because of the short distance for grain filling. Therefore, decreasing the rice grain width could promote rice AQ. Importantly, grain weight loss resulting from reduced grain width could be compensated by increasing the grain length. In conclusion, breeding elite rice with a slender grain shape is a practical strategy to improve rice quality without sacrificing its yield.

**Fig. 3:**
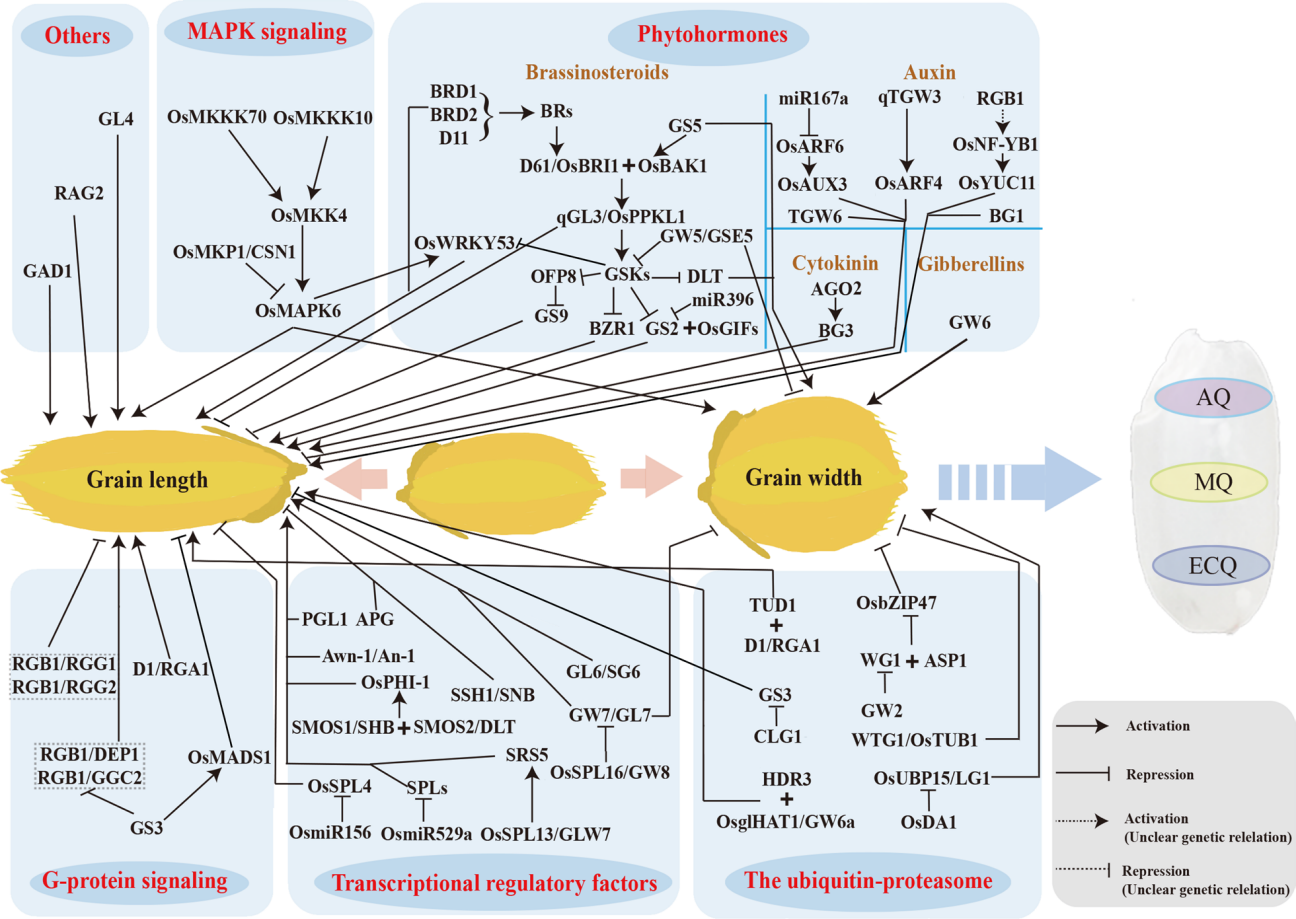
The cloned genes and the major regulatory networks controlling rice size. Rice seed size is regulated by multiple signaling pathways, including G protein pathways, the ubiquitin–proteasome pathway, MAPK signaling pathways, phytohormones, and transcription regulatory factors. A dotted line indicates that the genetic relationship needs to be further verified. MAPK, Mitogen-Activated Protein Kinase; AQ, appearance quality; MQ, milling quality; ECQ, eating and cooking quality

Therefore, the study of grain size has become a hot research topic among rice geneticists and breeders. At present, more than 400 quantitative trait loci (QTLs) linked to grain size have been mapped on all 12 chromosomes of rice, and over 80 grain size-related genes have been cloned (Huang et al. 2013; Zuo and Li 2014). Most importantly, several grain shape genes have been used in rice breeding practice, such as *GS3* and *DEP1/qPE9* (Huang et al. 2009; Zhou et al. 2009). This topic is well documented by several review papers (Li and Li 2016; Azizi et al. 2019; Li et al. 2019). Therefore, we have only summarized the functions and regulatory mechanisms of the key cloned genes related to grain size, especially the newly identified genes (Fig. 3).

### G Protein Pathways

The heterotrimeric G-protein complex consists of Gα, Gβ, and Gγ subunits. *GS3*, encoding a non-canonical Gγ subunit, is a main QTL controlling grain length and is a negative regulator of grain size (Fan et al. 2006). The *gs3* allele corresponds to long rice grains. The GS3 protein contains four functional domains, and the organ size regulation (OSR) domain is both necessary and sufficient to limit grain size (Mao et al. 2010). *DEP1/qPE9*, encoding another noncanonical Gγ subunit, is a major QTL for rice panicle architecture and grain size (Huang et al. 2009; Zhou et al. 2009). The *dep1/qpe9-1* allele, encoding a truncated protein, leads to erect panicles and smaller grains. Consistently, overexpression of *DEP1* increases the grain size, while knockout of the gene makes the grains smaller (Sun et al. 2018a). *DEP1/qPE9-1* regulates starch accumulation positively, mainly through promoting the expression of starch biosynthesis-related genes, thus prolonging the duration of the grain filling process, which finally affects the grain size (Zhang et al. 2012b).

In addition to GS3 and DEP1, the G protein γ subunits also include RGG1, RGG2 and GGC2. Grain size is regulated positively by GGC2 and negatively by RGG1 and RGG2 (Kato et al. 2004; Sun et al. 2018a; Miao et al. 2019; Xu et al. 2019). Recently, RGG1 was reported to be involved in regulating the cytokinin content, thus forming a G protein-cytokinin module to control rice grain size (Tao et al. 2020). In addition, genetic analysis indicated that these Gγ proteins require Gα (RGA1) and Gβ (RGB1) subunits to control grain length (Sun et al. 2018a). A recent report indicated that RGB1 not only controls the grain size, but also controls the grain filling process by regulating the expression of *OsNF-YB1*, encoding a critical downstream effector of RGB1. In addition, OsNF-YB1 directly interacts with the *OsYUC11* promoter to stimulate its expression, thus altering auxin homeostasis, starch biosynthesis and grain size (Zhang et al. 2021b).

### The Ubiquitin–Proteasome Pathway

The ubiquitin proteasome pathway (UPP) is an important system in eukaryotes that regulates protein stability and activity. *GW2*, a main QTL controlling grain width and weight, encodes a nuclear ring E3 ubiquitin ligase (Song et al. 2007). A loss of function allele of *GW2* promotes the proliferation of spikelet shell cells and produces wide grains. Importantly, this mutation notably enlarges the grain size and increases rice yield, but has little effect on rice AQ and ECQ. *Large grain1-D* (*lg1-D)* encodes a ubiquitin-specific protease 15 (OsUBP15). Loss-of-function of *OsUBP15* or suppressing its expression generates narrower rice seeds, while *OsUBP15* overexpression increases rice grain width significantly. Moreover, *OsUBP15* and *GW2* genetically interact with each other to co-regulate grain width (Shi et al. 2019).

*WG1* encodes a CC glutaredoxin that promotes grain growth by enhancing cell proliferation, while OsbZIP47 negatively regulates grain width and weight by suppressing cell proliferation. WG1 interacts with and inhibits the transcriptional activity of transcription factor OsbZIP47 by recruiting ASP1, a transcriptional co-repressor. Moreover, WG1 is ubiquitinated by E3 ubiquitin ligase GW2 and is subsequently degraded. Genetic analysis demonstrated that the three proteins form a GW2-WG1-OsbZIP47 molecular regulatory module to coordinate grain size and weight (Hao et al. 2021).

*TUD1* (*Taihu Dwarf1*) encodes a functional U-box E3 ubiquitin ligase, which interacts directly with D1/RGA1 to regulate plant height and grain size. In addition, *TUD1* and *D1* work together to regulate brassinosteroid (BR) signaling and produce short grains by reducing cell division (Hu et al. 2013). *OsTUB1/WIDE AND THICK GRAIN (WTG1)*, encoding a deubiquitinase, controls grain size by affecting cell proliferation. Knockout of *OsTUB1/WTG1* resulted in wider grains (Huang et al. 2017). In addition, two recent studies revealed novel regulators involved in UPP-mediated grain size regulation. One is HOMOLOG OF DA1 ON RICE CHROMOSOME 3 (HDR3), a ubiquitin interacting motif (UIM) type active ubiquitin receptor, can interact with and stabilize GW6a to slow down its degradation, thus promoting cell division and increasing the grain filling rate, which ultimately regulates grain size positively (Gao et al. 2021). GW6a is a histone acetyltransferase, whose overexpression increases grain weight and yield by increasing the cell number and accelerating grain filling (Gao et al. 2021). The other protein is Chang Li Geng1-1 (CLG1-1), an E3 ubiquitin ligase, which can ubiquitinate and mediate the degradation of GS3, thus changing G protein signaling and regulating the grain length (Yang et al. 2021b).

### Mitogen-Activated Protein Kinase (MAPK) Signaling

The MAPK cascade signaling pathway also plays an important role in regulating rice grain size. Typical MAPK pathways are usually composed of MAPKs, MAPK kinases (MKKs) and MKK kinases (MKKKs) (Zhang et al. 2018b). The OsMKKK10-OsMKK4-OsMAPK6 molecular cascade positively regulates rice grain size and weight (Xu et al. 2018). Disruption the expression of either member leads to smaller rice grains, while overexpression of these genes produces larger rice grains (Duan et al. 2014; Liu et al. 2015b). Furthermore, OsMAPK6 phosphorylates the transcription factor OsWRKY53 and enhances its activity (Tian et al. 2017), while GSK2 directly phosphorylates WRKY53 and lowers its stability (Tian et al. 2021).

In addition, OsMKKK70 also functions through the established OsMKK4–OsMAPK6–OsWRKY53 module (Liu et al. 2021). Overexpression of *OsMKKK70* leads to longer grain length and increased rice leaf angle. Moreover, overexpression of the genes encoding constitutively active OsMKK4, OsMAPK6, and OsWRKY53 in the context of *osmkk62/70* double mutation can partially restore its phenotype of grain size and leaf angle, implying that these elements operate in the same regulatory pathway.

*GSN1* encodes the mitogen activated protein kinase phosphatase, OsMKP1. Suppression of *GSN1* expression induces the proliferation of rice glume cells, resulting in larger but fewer rice grains. GSN1 directly interacts with and inactivates OsMAPK6 through dephosphorylation, thus playing an opposite role to OsMKK4 (Guo et al. 2018; Xu et al. 2018). Therefore, the key to controlling rice grain size is to accurately regulate OsMAPK6 activity through reversible phosphorylation.

### Phytohormone Perception and Homeostasis

Plant hormones, as central regulators of plant growth and development, not only orchestrating intrinsic developmental programs, but also conveying environmental inputs. Recently, a series of publications reported that some phytohormones, including BR, auxin, gibberellic acid (GA), and cytokinin, also play essential roles in regulating seed size via multiple molecular mechanisms.

#### BR is Involved in the Regulation of Grain Size

BRs, a group of plant-specific polyhydroxylated steroidal hormones, control a wide range of growth and developmental events, including grain size (Li et al. 2018b). Some BR mutants with defects in both BR biosynthesis or signaling, such as *brd1* (Hong et al. 2002; Mori et al. 2002), *dwaf2* (Hong et al. 2005), *dwaf11* (Tanabe et al. 2005; Zhu et al. 2015; Wu et al. 2016b; Zhou et al. 2017c), *OsBRI1/D61* (Yamamuro et al. 2000), *OsBAK1* (Li et al. 2009), *GS6/DLT/D62* (Tong et al. 2009; Sun et al. 2013), and *OsBZR1* (Zhu et al. 2015), usually exhibit shorter plants and smaller grains. One exception is GSK2, which, as a GSK3/SHAGGY like kinase homologous to Arabidopsis BIN2 in rice, is a central negative regulator of the BR pathway. Therefore, suppressing *GSK2* expression increased both grain size and leaf angles (Tong et al. 2012). Therefore, BR is a growth-promoting hormone with positive roles in regulating rice grain size.

*GW5* is a main QTL for grain width with three haplotypes (Zhou et al. 2017a). GW5 participates in the BR pathway and regulates grain width and weight by inhibiting the function of GSK2 kinase, thus releasing the active forms of OsBZR1 and DLT transcription factors (Liu et al. 2017). Meanwhile, *GW5* also affects rice quality, including the chalkiness rate, brown rice rate, and milled rice rate. *GS5*, encoding a serine carboxypeptidase (Yu et al. 2000), is a QTL controlling rice grain width. Increased expression of *GS5* inhibits the endocytosis of OsBAK1-7 and subsequently increases BR signaling and promotes grain size (Xu et al. 2015).

*GL3.1/GL3-1/qGL3/OsPPKl1* encodes a protein phosphatase OsPPKl1, which regulates rice grain length negatively by dephosphorylating OsGSK3 and inhibiting BR signaling (Hu et al. 2012; Qi et al. 2012; Zhang et al. 2012a; Gao et al. 2019). OsmiR396d regulates grain size by repressing the growth regulator GS2/GL2/OsGRF4, and GSK2 plays a similar inhibitory function, thereby affecting cell proliferation and grain shape (Che et al. 2015; Duan et al. 2015; Hu et al. 2015; Li et al. 2016; Sun et al. 2016). A recent study also showed that OsAGO17 forms an RNA-induced silencing complex (RISC) with OsmiR397b, which then affects rice development by inhibiting the expression of *OsLAC*, encoding a negative regulator of both grain size and grain number per panicle (Zhang et al. 2013; Zhong et al. 2020). Moreover, GSK2 interacts with and phosphorylates OML4, a negative regulator of grain size, thus modulating OML4 protein stability. Therefore, GSK2 and OML4 act in the same genetic pathway to regulate rice grain size (Lyu et al. 2020).

The mutation of *GS9*, encoding a novel transcriptional activator, leads to slender rice grains and reduced chalkiness, without affecting other major agronomic traits (Zhao et al. 2018). GS9 directly interacts with OVATE family proteins, thus forming a transcriptional complex to regulate glume cell division. Moreover, OFP8 and OFP14 inhibit the transcriptional activation activity of GS9, while OFP8 is directly suppressed by OsGSK2 in the BR pathway (Yang et al. 2016a). In addition to OFP8 and OFP14, several other OFP proteins also participate in grain size regulation via the BR pathway, including OFP1 (Xiao et al. 2017), OFP3 (Xiao et al. 2020), OFP19 (Yang et al. 2018a), and OFP22 (Chen et al. 2021). Among them, OFP1 is a positive regulator of BR signaling and seed size, while the others are all negative regulators.

Furthermore, two novel BR-related grain size genes were reported recently. One is *GW10*, encoding a P450 subfamily 89A2 homology protein, which plays a positive role in controlling grain size through the BR pathway (Zhan et al. 2021). The other is *POW1* (*Put On Weight 1*), encoding an unknown protein. The *pow1* mutant enhanced the endogenous BR content, leading to an enlarged leaf angle and grain size. Interestingly, downregulating the expression of BR biosynthesis or signaling genes could only restore the leaf angle phenotype of *pow1* mutant, not the grain size. Further analysis shows that POW1 regulates grain size by inhibiting the transactivation activity of TAF2, its interacting protein. Hence, two regulatory modules, POW1-TAF2 and POW1-BR, are established that specifically regulate the grain size and leaf angle, respectively (Zhang et al. 2021c).

#### Auxin

Auxin is an important classical phytohormone with essential roles in many aspects of plant growth and development events, including grain size. *qTGW6*, as a main QTL controlling rice grain weight, encodes indole-3-acetic acid (IAA)-glucose hydrolase to generate free IAA. A loss-of-function *TGW6* allele promoted grain length and weight (Ishimaru et al. 2013). Recent research indicated that *TGW6* is exclusively expressed in pre-emergent inflorescences, suggesting that TGW6 might play important roles in regulating pollen development (Akabane et al. 2021; Kabir and Nonhebel 2021).

*BG1*, an auxin primary response gene, encodes a protein that participates in the regulation of auxin transport. It affects grain size by promoting cell division and elongation (Liu et al. 2015a). *qTGW3/GL3.3* is a major QTL for grain weight, encoding a SHAGGY-like kinase 41 (OsSK41) (Hu et al. 2018; Xia et al. 2018; Ying et al. 2018). OsSK41 directly interacts with and phosphorylates OsARF4, a transcription repressor in the auxin pathway. Moreover, the OsSK41-OsARF4 module regulates rice grain size negatively by modulating the expression of a number of auxin-response genes. Recently, another transcription factor, OsARF6, was reported to bind directly to the *OsAUX3* promoter to increase its expression (Qiao et al. 2021). OsARF6 and OsAUX3 regulate rice grain length and weight negatively by modulating the auxin content and distribution in glume cells, consequently affecting rice grain longitudinal elongation. miR167a, a positive regulator of grain length and weight, directly silences *OsARF6* mRNA. Hence, a novel miR167a*-OsARF6*-*OsAUX3* regulatory module is established successfully.

#### GA

The tetracyclic diterpenoid phytohormone GA has multiple roles in plant growth and development. However, the participation of GA in regulating rice grain size is rarely reported. Recently, Shi et al. (2020) indicated that a QTL for grain size, designated as *GW6* (*GRAIN WIDTH 6*), was successfully cloned. *GW6*, encoding a GA-induced GAST family protein, plays positive roles in regulating grain size and weight. Moreover, knockout of *GW6* reduced the GA content in young panicles. Importantly, a natural variation in the CAAT-box of the *GW6* promoter determines its transcript abundance, as well as the grain width and weight, thus providing valuable natural genetic resources for rice breeding programs.

#### Cytokinin

A dominant mutant *big grain 3 (bg3-D)* was isolated, which featured larger rice grains. *BG3*, encoding a purine permease, OsPUP4, regulates grain size positively by controlling both the long-distance transport and local allocation of cytokinin (Xiao et al. 2019). Recently, Yin et al. (2020) reported that ARGONAUTE (AGO) proteins are essential to assemble RNA-induced silencing complexes to silence target genes. Overexpression of *AGO2* boosts both the salt-stress resistance and grain length of rice by modulating the histone methylation level of *BG3*, hence promoting its expression. Salt treatment results in a similar cytokinin distribution pattern to *AGO2* overexpression rice, implying that the cytokinin distribution pattern is critical to regulate stress tolerance and rice grain size.

### Transcription Factors

Transcription factors (TFs) play vital roles in regulating plant growth and development by responding to upstream signals and modulating downstream transcriptional networks. Certain TFs are also involved in controlling seed size.

#### SQUAMOSA Promoter Binding Protein-Like (SPL) Transcription Factor Family

SPL transcription factors are involved in controlling the tiller, panicle configuration, and grain size of rice. A genome-wide association analysis (GWAS) indicated that a major QTL *GLW7*, encoding the TF OsSPL13, is a key element leading to larger grains and more panicles (Si et al. 2016). GLW7 binds directly to the promoter of *SRS5*, a positive regulator of grain length, and activates its expression (Segami et al. 2017). Suppressing the expression of *OsSPL16*/*GW8* produces long grains, and thus decreased chalkiness and improved transparency of rice seeds (Wang et al. 2012). GW8 directly binds to the *GW7/GL7* promoter and inhibits its expression (Wang et al. 2015a). *GW7*, encoding a TONNEAU1-recruiting motif protein, plays a positive role in producing slender grains via differential regulation of cell division in both longitudinal and transverse directions. Importantly, a semi-dominant *GW7* allele enhances rice grain quality without any yield penalty (Wang et al. 2015c). Therefore, the identified OsSPL16/GW8-GW7 regulatory module should be useful in future elite rice breeding programs to improve both rice quality and yield. SPLs are known to be regulated by microRNAs, such as miR156 (Xie et al. 2006). Recently, two regulatory modules, miR529a-SPLs and OsmiR156-SPL4, are revealed and their essential roles in controlling rice grain size are studied (Hu et al. 2021; Yan et al. 2021).

#### APETALA2-Type (AP2) Transcription Factors

*SMOS1/SHB* encodes an APETALA2 (AP2) transcription factor with an incomplete AP2 domain. The grains and other organs of *smos1* mutants are smaller because of the smaller cells and an abnormal microtubule orientation. SMOS1 directly modulates the transcription of a cell expansion regulator, *phosphorylation inducible protein 1* (*OsPHI-1*) (Aya et al. 2014). SMOS1 interacts with SMOS2/DLT to form a key protein complex to orchestrate BR and auxin signaling, thereby coordinating rice growth and development, including grain size (Hirano et al. 2017). Moreover, *Suppression of Shattering 1* (*SSH1*) is a novel allele of *SUPERNUMERARY BRACT* (*SNB*), encoding an AP2 transcription factor. A point mutation in the ninth intron of *SNB* alters its mRNA splicing and decreases SNB expression, consequently reducing rice shattering and increasing grain size (Jiang et al. 2019).

#### Basic Helix-Loop-Helix (bHLH) Family

*Awn-1*(*An-1*) encodes a bHLH protein that regulates awn development, grain size, and grain number in rice. Increased *An-1* expression causes long awns and grains, but decreases the grain number per panicle (Luo et al. 2013). PGL1 is an atypical bHLH protein without DNA binding activity. Overexpression of *PGL1* increases the grain length and weight while, APG exerts an opposite effect. PGL1 interacts directly with APG to regulate grain size antagonistically (Heang and Sassa 2012).

#### Other Transcription Factors

*SHORT GRAIN 6 (SG6)/GL6* encodes a plant specific PLTAZ transcription factor which regulates grain length positively by promoting cell proliferation in young panicles and grains (Wang et al. 2019; Zhou and Xue 2020). GL6 interacts with RPC53 and OsTFC1 to participate in the RNA polymerase III transcription machinery and regulates the expression of genes involved in rice grain development and the cell cycle, thus regulates grain length positively (Wang et al. 2019). FLR family proteins, such as FLR1, FLR2, and FLR8, play negative roles, while FLR15 plays a positive role, in regulating grain size. Although the grains of the *flr8* mutant are larger, their quality remains the same. Moreover, *FLR1* can modulate the number of glume cells and the expression of starch metabolism genes, thus affecting seed size and grain filling (Wang et al. 2021). *qLGY3/OsLG3b*, a QTL for grain length, encodes the MADS-domain transcription factor, OsMADS1. *qLGY3/ OsLG3b* allele leads to the alternative splicing of *OsMADS1*, which is artificially selected and corresponds to a long grain phenotype (Liu et al. 2018b; Yu et al. 2018). GS3 and DEP1 directly interact with MADS1 to promote its transcriptional activity and hence inhibit grain growth (Liu et al. 2018b).

### Other Functional Proteins

*GAD1* encodes a small secretary signal peptide and its mutation leads to reduced grain numbers, shorter grains, and awnless rice. Mechanistically, GAD1 regulates the length of the grain and awn by modulating cell numbers (Jin et al. 2016). *GL4*, a QTL for grain length originating from African rice, encodes a Myb-like protein similar to SH4/SHA1. GL4 controls grain length by regulating the elongation of longitudinal cells of both the outer and inner glumes. A single nucleotide polymorphism (SNP) mutation in *GL4* leads to a premature stop codon and consequently a truncated protein, which results in small seeds and the loss of seed shattering during African rice domestication (Wu et al. 2017). In addition, overexpression of *RAG2*, encoding a 16-kDa α-amylase/trypsin inhibitor, significantly increases the grain size and 1000-grain weight, as well as the protein and total lipid contents (Zhou et al. 2017b).

## Genes Regulating Endosperm Components and Their Roles in Grain Quality

**Fig. 4:**
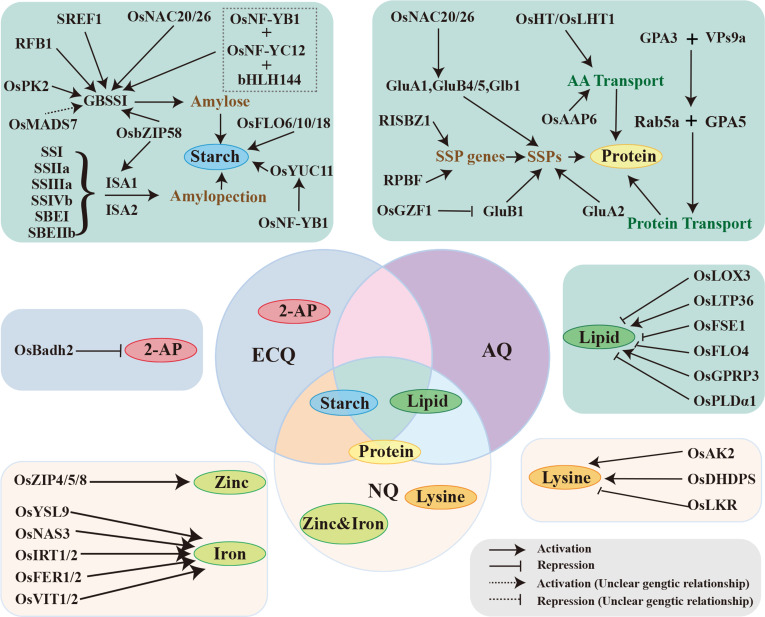
The genes involved in the synthesis and regulation of the main components of the endosperm, and their contributions to corresponding rice quality traits. The dotted line indicates that an indirect relationship that is supported by genetic evidence, which needs to be further verified. AQ, appearance quality; NQ, nutritional quality; ECQ, eating and cooking quality

The components of the rice endosperm include starch, proteins, amino acids, lipids, vitamins, minerals, and other metabolites. Among them, starch and protein account for about 80% and 10% of the dry weight of rice endosperm, respectively (Wang et al. 2020a). Therefore, the constitution and quality of starch and protein contribute majorly to rice grain quality (Fig. 4). In general, starch is the primary determinant of rice ECQ and AQ (Li et al. 2018a). For example, rice AC influences a series of quality related parameters, including hardness, viscosity, and transparency. Proteins are considered as the most important elements of rice NQ. Their content and quality determine rice NQ. Protein quality is mainly evaluated by assessing essential amino acids, such as lysine. In contrast to starch and protein, the content of lipid is low in rice, accounting for only about 0.3–0.6% of rice weight (Morrison 1988). The lipid content, similar to the protein content, correlates negatively with rice ECQ. Nevertheless, lipids have a great influence on the storage, processing, and consumption of rice. In addition to these three components, some micronutrients and phytochemicals are also important in rice quality, such as vitamins and minerals (Buttery et al. 1983; Kovach et al. 2009).

### Genes Controlling Starch Biosynthesis, Their Transcriptional Regulation, and Their Effects on Rice Quality

#### Starch Biosynthesis Enzymes

Starch is mainly stored in the form of starch granules in endosperm cells. According to their different glycosidic bond connections, starch is usually classified into two groups, amylose and amylopectin. The amylose content (AC) is the most important effector of rice ECQ (Duan and Sun 2005). According to their different ACs, rice varieties can be divided into waxy (< 2%), very low (3–9%), low (10–19%), intermediate (20–25%), and high (> 25%) amylose types (Zhang et al. 2017). In general, the higher the AC, the harder the texture of the cooked rice. Meanwhile, the AC is also related closely to rice transparency (Li et al. 2018a). Glutinous rice, lacking almost all amylose, has a completely opaque, waxy endosperm.

To date, almost all the genes encoding key enzymes involved in starch biosynthesis have been cloned and studied, such as ADP-glucose pyrophosphorylases (AGPases), granule-bound starch synthases (GBSSs), soluble starch synthases (SSSs), starch branching enzymes (SBEs), debranching enzymes (DBEs), and starch phosphorylase (PHO) (Zemach et al. 2010; Seung and Smith 2019). Detailed information related to starch biosynthesis is well summarized by some excellent reviews (Huang et al. 2020, 2021a); therefore, we will only focus on several key genes that determine rice quality.

AGPase uses glucose-1-phosphate (Glu-1-P) as a substrate to generate ADPglucose, which is then used to synthesize amylose via GBSSI, and to generate amylopectin via the cooperation of a series of other enzymes, including SSSs, SBEs, and DBEs (Jeon et al. 2010; Pfister and Zeeman 2016). Among them, GBSSI, encoded by the *Waxy* (*Wx*) gene, is the sole enzyme directly controlling amylose synthesis, and is thus the primary determinant of rice AC, GC, and pasting property (Wang et al. 2010). Therefore, *Wx* has been studied extensively and used widely to improve rice ECQ. Hence, a number of useful natural *Wx* alleles have been cloned and applied in rice breeding practice. Up to now, about ten natural *Wx* alleles have been reported, including the newly cloned *Wx*^*lv*^ and *Wx*^*mp*^/*Wx*^*la*^ (Zhang et al. 2019a, 2021a; Zhou et al. 2021a). The *wx* allele, a null allele that does not encode a functional GBSSI, exists in glutinous rice, AC < 2% (Wanchana et al. 2003). *Wx*^*a*^ and *Wx*^*b*^ are two major *Wx* alleles that are distributed widely in most indica rice and japonica rice varieties, respectively, corresponding to high and low ACs.

The discovery of the *Wx* ancestor gene, *Wx*^*lv*^, and the differentiation of its functional sites might explain the evolutionary trend of the AC, from high to low during rice domestication (Zhang et al. 2019a). The AC decreased slightly because of the variation in amino acid sequence caused by a SNP mutation in exon 6 of *Wx*^*in*^. Rice with the *Wx*^*op*^*/Wx*^*hp*^ (AC ~ 12.8%), *Wx*^*mp*^ (AC ~ 10.5%), and *Wx*^*mq*^ (AC ~ 10%) alleles are the so called soft rice (Hiroyuki et al. 2002; Mikami et al. 2008; Liu et al. 2009; Zhang et al. 2021a; Zhou et al. 2021a, b), which are famous for their good taste and high ECQ. The rare allele *Wx*^*mw*^*/Wx*^*la*^, derived from the homologous recombination of *Wx*^*i*n^ and *Wx*^*b*^, has a low AC, high transparency, good taste, and excellent ECQ (Zhang et al. 2021a; Zhou et al. 2021a). In addition to the identification of natural *Wx* alleles, the CRISPR/Cas9 gene editing strategy was also applied for accurate editing and to generate novel and excellent *Wx* alleles (Huang et al. 2020; Zeng et al. 2020).

The synthesis of amylopectin is complex and is coordinately regulated by several groups of enzymes. Moreover, each group contains several different types of enzymes. For example, SSS comprises SSSI, SSSII, SSSIII, and SSSIV. Except for SSSI, the other type of SSS all contain more than one isoform. Each enzyme isoform plays a distinct role in amylopectin biosynthesis. In general, SSSI prolongs the amylopectin by synthesizing short chains, while SSSII synthesizes medium-length amylopectin. *SSSIIa*/*ALK* is the major gene regulating rice GT (Gao et al. 2003). The different expression levels and allele types of *ALK* are the main cause for the differential amylopectin structure between indica and japonica subspecies (Umemoto et al. 2002). Two SNP-induced amino acid mutations affect the function of SSSIIa, resulting in a decrease in the branch chain length and GT (Bao et al. 2006). Recently, a detailed analysis of various *ALK* alleles, including a new identified *ALK*^*d*^ allele, was performed, which clarified their roles in regulating rice GT, AC, and general taste values, demonstrating *ALK* is a crucial molecular target to improve rice ECQ (Chen et al. 2020; Zhang et al. 2020). Another SSSII isoform, SSSII-2, was reported to have the potential to improve rice quality. Suppressing *SSSII-2* expression produced a novel soft rice with a low AC, improved taste, and transparent endosperm (Li et al. 2018a). Furthermore, simultaneous modulation of *SSSII-2*, *SSSIIa*, and *Wx* coordinated the biosynthesis of amylose and amylopectin, hence successfully improving rice ECQ (Huang et al. 2021b).

SSSIII synthesizes long amylopectin chains and SSSIIIa is an important target to study amylopectin biosynthesis and breed healthy rice. Resistant starch (RS) can reduce the incidence of type 2 diabetes and reduce the probability of obesity; therefore, high RS rice is considered to be a healthy food. SSIIIa affects the structure of amylopectin, the amylose content, and the physicochemical properties of starch grains in indica rice together with the *Wx*^*a*^ allele, resulting in a higher AC and an increased lipid content, subsequently increasing the amount of amylose–lipid complex and RS starch (Zhou et al.^2016^). That study suggested that modulating the *SSSIIIa* and *Wx* genes could benefit future breeding of high RS rice (Zhou et al. 2016).

#### Transcription Factors

The biosynthesis of starch is crucial to both seed development and propagation, and a number of transcription factors are involved in regulating the expression of starch synthesis-related genes (SSRGs). OsbZIP58/RISBZ1 binds specifically to the ACGT motif in the *Wx* gene promoter (Wang et al. 2013), thus enhancing its expression. In addition, OsbZIP58 also binds to the promoters of *AGPL3*, *SSSIIa*, *SBE1*, and *ISA2* to regulate their expression levels. bZIP58 is a core regulator of starch synthesis and its null mutant is chalky and its total starch and AC are decreased (Wang et al. 2013). In addition, OsbZIP58 interacts with RPBF, a Dof family transcription factor, to participate in the synthesis of storage substances, including starch, protein, and lipid, during rice endosperm development (Kawakatsu et al. 2009).

As an NF-Y transcription factor, OsNF-YB1 regulates endosperm sucrose transport and grain filling. Suppression of *OsNF-YB1* expression leads to development defects of rice seeds with increased grain chalkiness and a decreased AC, resulting in a decline in rice quality (Sun et al. 2014). Knockout of *OsNF-YB1* resulted in an increased protein content and decreased grain size and contents of amylose, total starch, crude fiber, and lipid, subsequently altering rice quality (Bello et al. 2019; Xu et al. 2021). In addition, OsNF-YB1 binds directly to the promoter of *OsYUC11* and activates its expression. As a key element in auxin biosynthesis, OsYUC11 affects grain filling and the accumulation of endosperm storage products in rice (Xu et al. 2021). Another NF-Y member, NF-YC12, coordinates various pathways to regulate endosperm development and the accumulation of seed storage substances in rice. The phenotype of the *osnf-yb12* mutant is similar to that of the *osnf-yb1* mutant, with changed grain weight, starch, and protein accumulations (Bello et al. 2019; Xiong et al. 2019). Furthermore, NF-YB1 combines with NF-YC12 and bHLH144 to form an NF-YB1-YC12-bHLH144 heterotrimeric complex that coordinates grain development and rice quality. Hence, the mutation of any gene in the complex would change starch synthesis in the rice endosperm (Bello et al. 2019).

Several members of MADS box family transcription factors participate in the regulation of starch biosynthesis. OsMADS6 is highly expressed in the endosperm and regulates the expression of SSRGs. Its mutation leads to decreased starch plumpness and abnormal endosperm development (Zhang et al. 2010). Suppression of *OsMADS29* expression caused abnormal seed development, such as shrunken seeds, a low grain-filling rate, and insufficient starch accumulation. Evidences indicates that OsMADS29 modulates the expression of genes related to programmed cell death (PCD), thus affecting the early development of rice seeds (Yin and Xue 2012). Another study revealed that OsMADS29 affects embryo and endosperm development, including starch biosynthesis, by modulating cytokinin signaling and biosynthesis (Nayar et al. 2013). As a high temperature induced gene, *OsMADS7* encodes a protein involved in stabilization of the AC in response to high temperature, mainly by enhancing the expression of GBSSI, the key enzyme controlling amylose biosynthesis. Therefore, OsMADS7 is a valuable molecular target for breeding elite rice with ideal thermal tolerance and ECQ (Zhang et al. 2018a).

NAC transcription factors are plant-specific and participate in various processes of plant development, including biosynthesis of storage substance of the rice endosperm. Mutation of *OsNAC20* or *OsNAC26* alone does not have any effect on rice grains. However, the contents of starch and storage proteins in *osnac20/26* double mutants are decreased. Further evidence demonstrated that OsNAC20 and OsNAC26 can promote the expression of multiple genes involved in starch and storage protein biosynthesis directly, such as those encoding SSSI, Pul, glutelin A1 (GluA1), glutelin B4/5 (GluB4/5), α globulin, and 16 kDa prolamin, thus regulating the synthesis of both starch and storage proteins (Wang et al. 2020a). ONAC127 and ONAC129 are not directly involved in starch synthesis in the rice endosperm. They regulate grain filling and starch accumulation by forming heterodimers, and participating in cytoplasmic transport and heat stress translation (Ren et al. 2021).

#### Other Proteins

In addition to the above mentioned starch synthesis-related enzymes and transcription factors, other proteins are involved in regulating starch biosynthesis. In general, the mutation of these genes, such as *FLO6* (Peng et al. 2014), *FLO10* (Wu et al. 2019)*, FLO14* (Xue et al. 2019)*, FLO18* (Yu et al. 2021), *FGR1* (Hao et al. 2019) and *OsPK2* (Cai et al. 2018), lead to defects in starch biosynthesis and the formation of abnormal starch granules, resulting in opaque, chalky, or powdery grains. Interestingly, only FLO6 shows a direct correlation with starch biosynthesis-related enzymes. FLO6 binds directly to starch through a CBM48 domain at the C terminus and to ISA1 through a domain at the N terminus, suggesting its role as a bridge between ISA1 and starch during starch synthesis (Peng et al. 2014).

### Storage Proteins, Transporters of Amino Acids and Proteins, and Other Regulators of Proteins

Proteins are the secondary major components of rice endosperm, which could be divided into three categories, storage proteins, structural proteins, and protective proteins. The protein content and its amino acid constitution affect rice NQ directly. Moreover, the protein content is also involved in the regulation of rice ECQ. In general, a negative correlation exists between the protein content and ECQ in rice (Hori et al. 2016).

#### Storage Proteins

The rice storage proteins (SSPs) can be divided into four categories, albumin, globulin, prolamine, and glutelin. Among them, glutelin, as the most abundant SSP, has the highest nutritional value because of its high digestibility and lysine content (He et al. 2021). There are 15 glutelin encoding genes in the rice genome, which are classified into four subfamilies, GluA, GluB, GluC, and GluD (Kawakatsu and Takaiwa 2010). Glu genes encode 57 kDa pro-glutelin, consisting of a signal peptide, a 37 kDa acidic subunit, and a 20 kDa basic subunit. A SNP in the *GluA2* promoter leads to a difference in the total protein content between indica and japonica rice. Hence, all haplotypes can be divided into two expression types, *OsGluA2*^*LET*^ and *OsGluA2*^*HET*^. *OsGluA2*^*LET*^ mainly exists in japonica rice, with low expression of OsGlu. Meanwhile, *OsGluA2*^*HET*^ is highly expressed in indica rice. Therefore, the expression of *OsGlu* genes correlate closely with the grain total protein content and NQ (Yang et al. 2019).

#### Transporters of Amino Acids and Proteins

Efficient amino acid transfer, depending on amino acid transporters (AATs), is essential for protein biosynthesis in rice grains. Lysine-Histidine-type Transporter 1 (OsLHT1) can transport a broad spectrum of amino acids effectively. *OsLHT1* mutation leads to declined root uptake and consequent transfer of amino acids to rice shoots (Guo et al. 2020b). Moreover, *Oslht1* mutant rice has a reduced panicle length, seed setting rate, grain number per panicle, and total grain weight. More N and free amino acids are retained in the flag leaf of the *Oslht1* mutant than in the wild-type at maturation, implying its essential roles in transferring amino acids from leaves to seeds, thus ensuring grain development and rice nutrition quality (Guo et al. 2020a). Furthermore, a number of amino acid permeases (AAPs), a main type of AAT, are responsible for amino acid loading in rice seeds. Mutation of *OsAAP10* decreases the content of both protein and amylose in rice seeds. In addition, the RVA profile of *osaap10* mutant seeds exhibits a higher peak viscosity, disintegration value, and lower recovery value, thus improving the ECQ (Wang et al. 2020b). GPA1/OsRab5a, a small GTPase, regulates the transport of glutelin to protein body II (PBII) and affects the protein content. GPA3 interacts directly with Rab5a and guanine exchange factor VPS9a, forming a regulatory complex with them. These three proteins regulate dense vesicle (DV)-mediated post-Golgi transport synergistically in rice. The *gpa3* mutant showed a powdery endosperm, abnormal accumulation of glutelin precursors, irregularly arranged starch grains, decreased amylose content, and increased levels protein and lipids (Ren et al. 2014). GPA5, an effector of Rab5a, is also required for post-Golgi trafficking of storage proteins (Ren et al. 2020). *GPA5* mutation also leads to a powdery white endosperm, resulting from the abnormal accumulation of glutelin precursors and a reduction in α globulin, consequently forming loosely arranged and round compound starch granules. GPA5 interacts with class C core vacuole/endosome tethering (CORVET) complex and VAMP727-containing soluble N-ethylmaleimide sensitive factor attachment protein receptor (SNARE) complex to promote the fusion of DVs and protein storage vacuoles to complete glutelin transportation.

#### Other Regulators

In addition to the above mentioned storage or functional proteins, other regulators also participate in regulating the protein content in rice endosperm. For example, RISBZ1/OsbZIP58 and RPBF regulate the expression of seed storage protein genes and the consequent protein content (Kawakatsu et al. 2009). OsGZF1, a CCCH type zinc finger protein, binds specifically to the core promoter region of *GluB-1*, thereby suppressing its expression and the accumulation of glutelins (Chen et al. 2014). Moreover, transcription factors NAC20 and NAC26 also bind directly to the promoters of *SSP* genes and regulate their expression in rice (Wang et al. 2020a). In addition to transcriptional regulation, control of glutelin mRNA localization is another important mechanism that modulates glutelin accumulation. The zip codes of storage protein mRNAs require assistance from RNA‐binding proteins (RBPs) for their correct localization. RBP‐A, RBP‐P, RBP‐L, and Tudor‐SN are reported to bind to the mRNAs of both glutelin and prolamin to aid their localization (Wang et al. 2008; Doroshenk et al. 2014; Chou et al. 2017, 2019; Tian et al. 2018; Tian et al. 2019a). A recent study showed that a quaternary protein complex, including RBP‐P, RBP‐L, Rab5a, and a membrane fusion protein NSF, cooperates to coordinate the transport of glutelin mRNAs in the rice endosperm (Tian et al. 2020).

### Lipids

The main fatty acids in rice are palmitic acid (C16:0), oleic acid (C18:1), and linoleic acid (C18:2). Among them, linoleic acid is relatively good for human health. Lipids not only affect the rice NQ, but also influence the AQ and ECQ. Phospholipids and glycolipids can interact with starch in rice, thus reducing the water absorption and expansibility of starch, and can increase its GT.

The genes involved in carbon flow, lipid biosynthesis, transport, and oxidation affect the quantity and quality of lipids in rice. Pyruvate phosphate dikinase (encoded by *OsPPDKB)* regulates both carbon metabolism and carbon flow for starch and fat biosynthesis during rice filling. Its mutation produces a white powdery endosperm and a significantly increased fat content (Zhao et al. 2017). Another gene *Floury Shrunken Endosperm1* (*FSE1*), encoding a phospholipase-like protein, regulates galactolipid biosynthesis in the rice endosperm. *FSE1* mutation not only reduced the total galactolipid content significantly, but also caused abnormal amyloplast development in the developing endosperm, thus providing a novel connection between lipid metabolism and starch synthesis in rice (Long et al. 2018).

Fatty acid desaturase (*FAD*) genes, including *OsFAD2* and *OsFAD3*, participate directly in different steps of fatty acid synthesis (Liu et al. 2012; Shi et al. 2012; Ding et al. 2015). *OsACOT*, a major target of miR1432-mediated cleavage, encodes acyl-CoA thioesterase, which is involved in the biosynthesis of medium-chain fatty acids. Suppression of miR1432 expression or overexpression of miR1432-resistant form of *OsACOT* (*OXmACOT*) promoted rice grain filling rate and grain weight significantly. Moreover, the contents of palmitic acid and stearic acid (18:0) in *OXmACOT* transgenic rice decreased, while those of the oleic acid and linoleic acid increased (Zhao et al. 2019).

Suppressing the expression of *OsLTP36*, encoding a lipid transporter, resulted in decreased contents of fatty acids and proteins, smaller and loose starch grains, and some other growth defects, including the seed setting rate, 1000-grain weight, chalkiness, and seed germination rate (Wang et al. 2015b). Moreover, lipoxygenase (LOX) catalyzes lipid oxidation, which leads to aging and a decrease of nutrient level in rice (Cho and Lim 2016). *LOX-2* and *LOX-3* regulate the degradation of fatty acids negatively, and suppression of their expression or loss-of-function mutation effectively prolonged the storage time and maintained a high nutritional value of rice (Long et al. 2013). Meanwhile, reducing the expression of *LOX-3* could effectively reduce the degradation of β-carotene in golden rice (Huang et al. 2014; Zhou et al. 2014). A recent study showed that mutation of *OsPLDα1*, encoding a phospholipase, changed lipid metabolites and reduced the phytic acid content strikingly (Khan et al. 2019). Further analysis indicated that the mutant brown rice shows some changes in ECQ properties, including a decreased AC, setback viscosity, and gelatinization temperature (GT), as well as an increased disintegration rate, corresponding to improved ECQ and NQ (Khan et al. 2020).

### Lysine

The types of amino acids and the proportion of essential amino acids also determine the nutritional quality of rice. Lysine (Lys) is considered as the first limiting essential amino acid in humans; however, its content in milled rice is quite low. Several strategies have been used to enhance the lysine content in rice, including overexpression of lysine-rich proteins and modulation of lysine metabolism pathways (Yang et al. 2021a). For example, overexpression of lysine-rich histone proteins, RLRH1 and RLRH2, increased the lysine content of rice by 35% (Wong et al. 2015). With regards to modulation of lysine metabolism pathway, one method is to enhance the expression of lysine biosynthesis genes, another is to block the catabolism of lysine, thus promoting the lysine content of rice. AK and DHPS, two rate-limiting enzymes in the lysine biosynthesis pathway, are under strict feedback inhibition by lysine. By overexpressing modified Lys-insensitive AK or DHPS, the free lysine content increase by 6.6- to 21.7-fold. When simultaneously expressing these two enzymes, the level of free lysine increased by 58.5-fold (Yang et al. 2020, 2021a). Suppressing the expression of *LKR/SDH* gene, encoding lysine ketoglutaric acid reductase/saccharopine dehydropine dehydrogenase (LKR/SDH), attenuated lysine catabolism and remarkably promoted the free lysine content in rice (Wu et al. 2016a; Yang et al. 2016b; Yang et al. 2018b; Zheng and Wang 2014). The rice varieties with high free lysine content had no significant differences in yield and other main agronomic traits except for plant height and grain color (Yang et al. 2016b). The changed endosperm color of high-lysine rice is mainly caused by activation of the jasmonic acid pathway by the high abundance of free lysine and subsequent enhanced serotonin biosynthesis (Yang et al. 2018b).

### Carotenoids

Regardless of the fact that more than 700 kinds of carotene have been found in nature, only α-carotene, β-carotene, lutein, lycopene, zeaxanthin, and astaxanthin have been shown to be beneficial for health (Federico and Schmidt 2016). Carotenoids are important phytonutrients with antioxidant properties, and are used widely in foods and feedstuffs as supplements. In addition, carotenoids can also be used as antioxidants to prevent seed aging and promote seed vigor, leading to successful germination (Federico and Schmidt 2016). The synthesis of carotenoids in seeds is closely related to the ABA biosynthesis pathway, the dominant pathway for seed dormancy.

Rice carotenoids biosynthesis is blocked in the first enzymatic step. Biofortification is an effective way to produce and accumulate carotenoids in rice grains. Therefore, the major objective of golden rice (GR) development is to improve its carotenoids content. Driven by the *Gt1* promoter, the daffodils-originating *PSY* gene and *Erwinia uredovora*-originating *CRTI* gene were transformed into rice, thus generating rice grains with β-carotene accumulation (Ye et al. 2000). Golden rice 2 (GR2) was developed by transferring the maize phytene synthase gene *Zmpac1* and the carotene desaturase gene *CrtI* from soil bacteria *Pantoea ananatis* into japonica Kaybonnet rice. The content of β-carotene in the rice grain was 23 times higher than that in the first generation of GR (Paine et al. 2005). Recently, astaxanthin biosynthesis was bioengineered in the rice endosperm by introducing *sZmPSY1*, *sPaCrtI*, *sCrBKT*, and *sHpBHY*, four genes encoding the enzymes phytoene synthase, phytoene desaturase, β-carotene ketolase, and β-carotene hydroxylase, respectively, thus generating multiple healthy rice germplasms, including β-carotene-enriched Golden Rice, Canthaxanthin Rice, and Astaxanthin Rice (Zhu et al. 2018). Another study indicated that co-expression of *tHMG1*, *ZmPSY1*, and *PaCRTI* could boost the carotenoids flux through the MVA pathway, thus increasing the accumulation of carotenoids in the rice endosperm markedly (Tian et al. 2019b). The promotion of carotenoids in rice relies on insertions of target genes at random sites via conventional transgenic methods; therefore, the marker gene still exists in the genome of transgenic rice. Alternatively, an expression cassette including two carotenoid biosynthesis genes were introduced using targeted insertion at a safe site in the rice genome via CRISPR/Cas9, thus generating marker-free carotenoid-enriched rice (Dong et al. 2020).

### Minerals, Taking Fe and Zn as Examples

At present, more than 90 Fe-related QTLs have been identified in the rice genome, among which 17 are stable and 25 harbor Fe-related genes nearby or within the QTL (Swamy et al. 2021). A common mechanism of transporters and chelators mediates iron and zinc absorption and transport; therefore, most instances of increased iron content in rice are accompanied by a parallel increase in zinc (Kawakami and Bhullar 2018).

There are two sources of iron in seeds, absorption by the roots from soil solution, followed by direct transfer to seeds and reactivation from different tissues and organs during seed development (Ishimaru et al. 2006, 2007a). Two Ferritin (FER) genes, *OsFER1* and *OsFER2*, have been identified in rice (Stein et al. 2009). *OsFER2* is more sensitive to external iron supply than *OsFER1,* implying its major role in rice FE chelation. Seed-specific overexpression of *OsFER2* promotes the accumulation of iron and zinc in milled rice seeds by 2.1 and 1.37-fold, respectively (Paul et al. 2012).

Nicotinamide (NA) is a ubiquitous metal-chelated non-protein amino acid in terrestrial plants with important role in metal transport. Increasing the expression of the NA synthase gene (*NAS*) is a useful biofortification method to promote the Fe and Zn contents of rice. There are three *NAS* genes in rice, *OsNAS1*, *OsNAS2*, and *OsNAS3* (Nozoye et al. 2019). Knockout of *OsNAS3* reduced the iron content in rice flag leaves and seeds, while *OsNAS3* overexpression had the opposite phenotype (Lee et al. 2009). Yellow Stripe 1-Like 9 (encoded by *OsYSL9*) transports the iron-sodium/DMA complex from the endosperm to the embryo during seed development. An *OsYSL9* null mutant showed a decreased iron content in embryos, but an increased iron content in the endosperm (Senoura et al. 2017).

OsVIT1 and OsVIT2 are another two transporters that regulate the iron content in rice seeds. Knockout of the two genes promotes the amount of Fe/Zn in rice seeds, but decreases contents of these metals in rice leaves, suggesting that OsVIT1 and OsVIT2 play important roles in controlling Fe/Zn translocation between source and sink organs (Zhang et al. 2012b).

ZIP transporters include zinc regulated transporters (ZRT) and iron regulated transporters (IRT). The rice *ZIP* gene family contains 16 members, including 14 *ZRT* genes and two *IRT* genes (Sasaki et al. 2015). Overexpression of *OsIRT1* reduces plant height, tiller, and yield of rice, but increases the content of Fe and Zn in rice grains (Lee and An 2009). In addition, overexpression of *OsZIPs*, such as *OsZIP4*, *OsZIP5*, and *OsZIP8*, promotes the Zn content in roots, but reduces the content in shoots and grains (Ishimaru et al. 2007b; Lee et al. 2010a, b).

In general, three major strategies can be used to enhance the iron content in rice, including overexpression of *NAS* genes, endosperm-specific expression of *FERs*, and promoting source-to-endosperm Fe remobilization (Kawakami and Bhullar 2021).

### 2-Acetyl-1-Pyrroline (2-AP)

2-AP is the main aroma substance in scented rice (Kovach et al. 2009; Bradbury et al. 2010). The *Badh2* gene, encoding betaine aldehyde dehydrogenase, inhibits the biosynthesis of 2-AP by exhausting γ-aminobutyraldehyde (AB-ald), a presumed 2AP precursor. The significant increase of 2-AP levels in fragrant rice varieties greatly improves the aroma of milled rice. The null *badh2* alleles, with a protein frameshift mutation, enhance 2-AP biosynthesis and hence the aroma of rice (Chen et al. 2008).

## Genes Regulating Other Seed Structures and Their Roles in Grain Qualities

### The Embryo

The rice embryo, containing most of the genetic information of rice, has the highest concentration of nutrients, including proteins, fatty acids, vitamins, and minerals. Giant embryo rice is a rice mutant whose embryo is about two to three times larger than the normal embryo. The phenotype of giant embryo rice is determined by the *GIANT EMBRYO* (*GE*) gene, encoding cytochrome P450 protein CYP78A13. *GE* mutation leads to large embryo and small endosperm, while overexpression of *GE* had the opposite phenotype (Nagasawa et al. 2013).

In plants, GABA is mainly catalyzed by glutamate decarboxylase (GAD), producing decarboxylate glutamate (Akama et al. 2009; Shelp et al. 2012). However, the higher GABA content in the grains of developing giant embryo rice mainly comes from the upregulation of the polyamine (PA) derivative pathway and the downregulation of GABA catabolism activity (Zhao et al. 2017). The C-terminal extension of OsGAD2 acts as a powerful self-inhibitory domain, and truncation of this domain caused the enzyme to function constitutively and actively. Rice endosperm specific expression of *OsGAD2ΔC* enhanced the GABA content by about tenfold (Shelp et al. 2012). In addition, the combination of seed-specific overexpression of truncated GAD with suppression of GABA-T, enhanced the GABA content strikingly (75–350 mg/100 g) in milled rice (Shimajiri et al. 2013). Recently, the coding region of CaMBD from the *OsGAD3* gene was knocked out using CRISPR/Cas9 technology, thus improving rice GABA content by 7 times, accompanied by increased grain weight and protein content (Akama et al. 2020).

### The Aleurone Layer

Brown rice is unpolished rice after shelling, which is composed of the endosperm, embryo, and rice bran. Compared with milled rice, brown rice contains more macronutrients, vitamins, minerals, and other functional substances, such as GABA, is thus more beneficial to human health. The proportion of nutrients among various rice components are different, thus optimization of rice structure is expected to change the nutritional quality of rice (Zheng and Wang 2014). Most nutrients of the seeds are stored in the endosperm. The triploid endosperm of rice develops from the fertilized polar nucleus. The endosperm at the filling stage consists of the aleurone layer, subaleurone layer, and starch endosperm, from outside to inside, respectively (Wu et al. 2016a). The cells of the aleurone layer of the mature endosperm are living, whereas the cells in the starch endosperm are dead. As a transitional cell type, the subaleurone layer cells accumulate both starch and protein in the early stage of development and differentiate into the starch endosperm in the late stage of endosperm development. The aleurone layer contains a large number of nutrient elements, such as proteins, vitamins, and minerals (Becraft and Yi 2011).

*TA1*, encoding a single-stranded DNA binding protein, OsmtSSB1 (located in mitochondria), is highly expressed in the aleurone layer, subaleurone layer and the caryopsis embryo, but not in the starch endosperm. The thickness of the aleurone layer of the mutant *ta1* was about twice as thick as that of the wild-type, and the nutritional quality was greatly improved. In the *ta1* mutant, the content of all nutrient elements increased (Li et al. 2021). In the mutant *ta2-1*, its grain transparency, 1000-grain weight, seed setting rate, total starch and AC were decreased; however, its nutritional composition of shelled grains increased, including the total protein, lipid, iron, zinc, calcium, dietary fiber, antioxidants, phenols, and vitamins (Liu et al. 2018a).

Although brown rice is rich in nutrients, it is accepted by few consumers in the market because of its poor taste. How to balance taste and nutrition is an urgent problem that should be solved in the future. Currently, using germinated brown rice seems to be a reasonable alternative, which maintains the nutritional quality of rice but improves its edible quality (Cho and Lim 2016).

### The Seed Coat

Brown rice or dehulled rice refers to rice with the hull removed, and further polishing generates milled white rice, which is generally sold in the market and consumed by people. However, brown rice contains most of the nutritional substances, including dietary fiber, vitamins, and phenolics, most of which are lost during the polishing process. Therefore, brown rice, as the whole grain, contains more nutritional components. Moreover, brown rice with a red, purple, or black pericarp is more beneficial to human health than traditional white pericarp rice because of the accumulation of more antioxidant compounds. Red rice pigmentation is controlled by the joint action of two genes *Rc* and *Rd* (Sweeney et al. 2006; Furukawa et al. 2007). *Rc*, encoding a basic helix ring helix (bHLH) transcription factor, is responsible for the accumulation of the pigment, and *Rd*/*OsDFR*, encoding a dihydroflavonol 4-reductase, enhances the accumulation of procyanidin in the brown grain pericarp (Sweeney et al. 2006; Furukawa et al. 2007). *Rc* and *Rd* together cause the peel to be red and most cultivated rice varieties that produce white grains have a 14 bp frameshift deletion of the seventh exon of *Rc* (Furukawa et al. 2007). Recently, the recessive *Rc* allele with 14 bp deletion was functionally restored to an in-frame mutation via the CRISPR/Cas9 method, which successfully transformed three excellent white grain varieties into red grains, thus remarkably promoting the content of procyanidin and anthocyanin (Zhu et al. 2019).

The black rice pigment is also regulated by the joint action of two genes, *Pb* and *Pp* (Hu et al. 1996; Wang and Shu 2007). The *Pb* locus is composed of two genes, encoding a MYC transcription factor and a bHLH16 transcription factor, which are involved in the synthesis of anthocyanin and procyanidins, respectively. The expression of *Pb* seems to be the cause of pigment accumulation in the pericarp of brown grains, whereas the expression of *Pp* increases the content of pigment, resulting in purple grains. The copy number of the *Pp* gene correlates with the intensity of purple pigmentation. In the absence of *Pp*, *Pb* plants produce brown grains, while *Pp* plants without *Pb* have white grains (Tanaka et al. 2008; Rahman et al. 2013; Mbanjo et al. 2020).

The gene corresponding to black rice is *Kala4*/*OsB2*, encoding a bHLH transcription factor. The structural rearrangement of its promoter region leads to its ectopic expression, resulting in a black pericarp. *OsB2* regulates a number of genes encoding anthocyanin synthesis related enzymes, including *F3H*, *DFR*, and *ANS* (Oikawa et al. 2015).

Recently, a *C-S-A* gene model of rice husk pigmentation was proposed. *C1* and *A1* jointly determine the color change, while *S1* diversifies the pigment tissue. *C1* encodes an R2R3-MYB transcription factor and acts as a color producing gene. *S1* encodes a bHLH protein that functions in a tissue-specific manner. C1 interacts with S1 and activates the expression of *A1*, which encodes dihydroflavonol reductase, thus generating purple rice pigment. The involvement of functional A1 leads to high accumulation of cyano 3-o-glucoside, while A1 mutation results in a brown pigment. Instead of C1, rice pigment color is produced by the synergistic regulation of S1 and other MYB transcription factors (Sun et al. 2018b).

More recently, a study showed that *OsTTG1*, the *WD40* gene in rice, is an important regulator of anthocyanin biosynthesis. The OsTTG1 protein is located in the nucleus and directly interacts with Kala4, OsC1, OsDFR, and Rc, which are determinants of pigments or anthocyanin biosynthesis. Knockout of *OsTTG1* reduced the anthocyanin content of the mutant grain to only 0.15% of that of wild-type plants, suggesting that OsTTG1 is a vital regulator of rice anthocyanin biosynthesis (Yang et al. 2021c).

## Conclusion and Perspectives

Rice grain quality, a typical quantitative trait, is influenced by complex genetic regulation and environmental factors. Rice quality generally includes milling quality, appearance quality, nutritional quality, and eating and cooking quality. The structure and composition of the rice seed are correlate closely with different aspects of rice quality. For example, grain size not only affects rice AQ, but also influences rice MQ and ECQ. Moreover, the rice endosperm, as the edible part of rice, is composed of starch, protein, lipids, and other micronutrients. The quantity and quality of these component are major contributors to rice ECQ, AQ, and NQ. Therefore, modifying and optimizing the structure and composition of rice seed are crucial to promoting its quality. Considering that rice growth and development are controlled by numerous genes and complicated regulation networks, the cloning of key genes involved in regulating specific grain trait and the dissection their molecular mechanisms will provide valuable gene resources and essential information for breeding high quality rice. In the present review, we summarized the cloned genes and their molecular functions that determine each part of rice seeds and their corresponding roles in regulating rice quality. In particular, the genes involved in controlling seed size and endosperm components are highlighted, which are also the two major determinants of rice quality.

Although impressive progress has been made in rice quality research, few cloned genes are suitable for use in high quality rice breeding programs. In addition, a huge gap still exists in our understanding of the regulatory network of rice grain quality. Therefore, a number of urgent problems remain to be solved. First, there is a contradiction between grain yield and rice quality; therefore, how to improve rice quality without sacrificing rice yield is an important issue. Second, it is a great challenge to explore the upstream and downstream components of known rice quality regulators, as well as the crosstalk between various regulation pathways. Third, the evaluation of the effects of most cloned rice quality genes or their elite alleles are limited and fragmented, being based on the analysis in a only a few rice varieties, especially some so-called model varieties. The performance of quality traits are genetic and environment-dependent, which often causes the silencing of some reported high quality genes in breeding practice. Fourth, some rice quality determinants often play contradictory roles in the context of different quality traits. For example, the protein content correlates positively with rice NQ. However, the higher the protein content, the more protein bodies accumulate among starch grains, resulting in increased chalkiness and the deterioration of ECQ. For lipids, the oxidation of unsaturated fatty acids helps to improve the flavor of rice. However, it is not conducive to long-term storage because the hydrolysis and oxidation of lipids will worsen the appearance and taste of rice. Finally, we lack universal and unified rice quality evaluation standards. Rice quality is relative and context-specific; thus the evaluation of rice quality depends on their application mode and consumer background, including region, age, or even religion.

Although rice quality research remains challenging, certain strategies should be employed in future studies. Most importantly, more genes encoding proteins with specific roles in the control of rice quality should be cloned, sequenced, and functionally analyzed, thus enlarging the high quality gene pool. In addition, powerful and accurate technologies can be used in basic research and in breeding practices of high quality rice, such as genome-wide selection, enhanced selection of major genes for grain quality, and precise gene knock-in or knock-out. For example, based on re-sequencing data of 200 japonica rice varieties in central China, Xiao et al. (2021) revealed a number of superior alleles for rice quality and rice blast resistance by using genome-wide association mapping and selection approach. Next, the alleles related to blast-resistance and excellent rice ECQ, such as *Wx*^*mp*^ allele, corresponding to low amylose content, were successfully introduced into two high-yield rice cultivars. Hence, two elite rice lines, XY99 and JXY1, with both ECQ and blast resistance improved were efficiently developed (Xiao et al. 2021). In addition, the corresponding breeding character targets, as well as universal and objective rice quality evaluation standards according to regional or population preferences of consumers, should be established as soon as possible, which will help to accelerate high quality rice research.

## Data Availability

Not applicable.

## Abbreviations

AAP10: Amino acid permease 10
ABA: Abscisic acid
AC: Amylose content
ACOT: Acyl-CoA thioesterase
AGPases: ADP-glucose pyrophosphorylases
AK2: Aspartate kinase 2
ALK: Alkali digestion
AQ: Appearance quality
AP2: APETALA2
Badh2: Betaine aldehyde dehydrogenase 2
BAK1: BRI1-ASSOCIATED RECEPTOR KINASE
BG3: Big grain 3
BR: Brassinosteroid
BRD1: BR-deficient dwarf1
BRI1: BRASSINOSTEROID-INSENSITIVE 1
BZR1: BRASSINAZOLE-RESISTANT 1
CaMBD: Calmodulin-binding domain
CLG1-1: Chang Li Geng1-1
CORVET: The class C core vacuole/endosome tethering
D1: Dwarf 1
DBE: Debranching enzyme
DEP1: Dense and erect panicle 1
DHPS: Dihydrodipicolinate synthase
DLT: Dwarf and low-tillering
ECQ: Eating and cooking quality
FAD: Fatty acid desaturase
FER1: Ferritin gene 1
FGR1: Floury and growth retardation 1
FLO6: FLOURY ENDOSPERM 6
FLR: FERONIA-like receptor
FSE1: Floury Shrunken Endosperm1
GA: Gibberellin
GABA: γ-Aminobutyric acid
GAD: Glutamate decarboxylase
GAD1: Grain length and awn development 1
GBSS: Granule-bound starch synthase
GC: Gel consistency
GE: GIANT EMBRYO
GGC: G protein gamma subunit
GL3.1: Grain length 3.1
GluA1: Glutelin type-A1
GPA1: GLUTELIN PRECURSOR ACCUMULATION1
GR: Golden rice
GRF4: Growth-regulating factor 4
GS3: QTL for grain size on chromosome 3
GS5: Grain size on chromosome 5
GS9: Grain shape gene on chromosome 9
GSK2: GSK3/SHAGGY-like kinase 2
GSN1: Grain size and number1
GT: Gelatinization temperature
GW2: Grain weight and width on chromosome 2
GW5: QTL for grain width and weight on chromosome 5
GW6: Grain width 6
GW6a: Grain weight on chromosome 6-a
GZF1: GluB-1-binding Zinc Finger 1
HDR3: Homolog of DA1 on rice chromosome 3
IRT: Iron regulated transporters
ISA: Isoamylase
LAC: Laccase
LG1: Large grain 1
LHT1: Lysine-histidine-type transporter 1
LKR: Lysine ketoglutaric acid reductase
LOX-2: Lipoxygenase 2
Lys: Lysine
MADS6: MADS-box transcription factor 6
MAPK: Mitogen-activated protein kinase
MKKs: MAPK kinases
MKKKs: MKK kinases
MQ: Milling quality
NAC20: NAC domain-containing protein 20
NAS: Nicotinamide synthase
NF-YB1: Nuclear transcription factor Y subunit B1
NQ: Nutrition quality
OFP: OVATE family protein
PA: Polyamine
PGL1: Positive regulator of grain length 1
PHI-1: Phosphorylation inducible protein 1
PHO: Starch phosphorylase
pk2: Plastidic pyruvate kinase 2
PLDα1: Phospholipase Dα 1
POW1: Put on weight 1
PPDKB: Pyruvate phosphate dikinase
PPKL1: Protein phosphatase with Kelch-like repeat domain 1
QTL: Quantitative trait locus
Rab5a: Small GTPase RAT BRAIN 5a
RBP: RNA‐binding protein
RGA: G protein alpha subunit
RGB: G protein beta subunit
RGG: Heterotrimeric G protein γ subunit
RISC: RNA-induced silencing complex
RLRH1: Rice lysine rich histone proteins 1
RVA: Rapid visco analyzer
SBE: Starch branching enzyme
SDH: Saccharo-pine dehydropine dehydrogenase
SMOS1: Small organ size 1
SNARE: Soluble N-ethylmaleimide sensitive factor attachment protein receptor
SNB: SUPERNUMERARY BRACT
SPL: SQUAMOSA promoter binding protein-like
SRS5: Small and round seed 5
SSH1: Suppression of shattering 1
SSRGs: Starch synthesis related genes
SSS: Soluble starch synthase
TA1: Thick aleurone 1
TF: Transcription factor
TGW6: Thousand-grain weight 6
TTG1: TRANSPARENT TESTA GLABRA1
TUD1: Taihu Dwarf 1
UBP15: Ubiquitin-specific protease 15
UPP: Ubiquitin proteasome pathway
VIT1: Vacuolar iron transporter 1
WTG1: Wide and thick grain 1
Wx: Waxy
YSL9: Yellow stripe1-like 9
YUC11: YUCCA (YUC) flavin-containing monooxygenase 11
ZRT: Zinc regulated transporters
